# Artificial Intelligence and AI-Adjacent Digital Technologies in Nursing Practice Within Gastroenterology: A Scoping Review of Current Applications, Challenges, and Future Directions

**DOI:** 10.3390/nursrep16100357

**Published:** 2026-10-10

**Authors:** Sonia Barros, Ana Carvalho, Guilherme Macedo, Joao C. Ferreira, Miguel Mascarenhas

**Affiliations:** 1 Department of Gastroenterology, São João Hospital, 4200-319 Porto, Portugal; sonia.barros@ulssjoao.min-saude.pt (S.B.); ana.veloso@ulssjoao.min-saude.pt (A.C.); guilhermemacedo59@gmail.com (G.M.); 2 Department of Community Medicine, Information and Health Decision Sciences (MEDCIDS), Faculty of Medicine, University of Porto, 4099-002 Porto, Portugal; miguelmascarenhassaraiva@gmail.com; 3 Faculty of Logistics, Molde University College, NO-6410 Molde, Norway; 4 Center for Research of Technologies and Architecture, Information Sciences, Technologies and Architecture Research Centre (ISTAR), Instituto Universitario de Lisboa ISCTE-IUL, 1649-026 Lisbon, Portugal

**Keywords:** artificial intelligence, AI-adjacent digital technologies, nursing, gastroenterology, scoping review, clinical applications

## Abstract

**Background/Objectives:** Artificial intelligence (AI) is increasingly integrated into clinical gastroenterology, with emerging applications in nursing contexts. However, the extent and nature of these applications in gastroenterology nursing remain under-explored. This scoping review aimed to map the current landscape of AI and closely related AI-adjacent digital technologies in gastroenterology nursing. **Methods:** Following the Preferred Reporting Items for Systematic Reviews and Meta-Analyses extension for Scoping Reviews (PRISMA-ScR) guideline, PubMed, Medline, Web of Science, and Scopus were searched for peer-reviewed, English-language studies published since 2000, and the findings were synthesized narratively. **Results:** Of 184 records identified, 11 studies were included, highlighting the potential of these technologies in clinical decision support, workflow optimization, patient education, and endoscopy assistance. Reported benefits include gains in workflow efficiency, technical accuracy, and reduced administrative workload; direct evidence of improved nursing-led patient outcomes was reported in only a minority of studies. Challenges encompass limited nurse-led research, insufficient external validation, implementation gaps, and ethical concerns. **Conclusions:** Greater nurse involvement in AI development is essential to drive paradigm shifts in clinical practice. This review synthesizes evidence to inform future applications, research, and policy in AI-enhanced gastroenterology nursing.

## 1. Introduction

Artificial intelligence (AI) is increasingly applied across the healthcare sector, with reported uses spanning patient care, clinical decision-making, and management processes [1]. AI technologies, including predictive analytics, machine learning, and natural language processing, have been associated with the potential to support patient outcomes, workflow optimization, and clinical decision-making [1,2,3].

Within gastroenterology, AI is increasingly utilized, particularly in the field of endoscopy. Algorithms have demonstrated efficacy in reducing human operator variability and improving procedural outcomes [4,5]. AI systems are widely recognized for their capacity to aid in image analysis and diagnosis, making gastroenterology a leading domain for AI adoption [6,7]. Although AI’s use in gastroenterology is well-documented, its direct integration into nursing practice within this specialty is less thoroughly studied. Nonetheless, broader research on AI in nursing provides insight into its potential applications and challenges relevant to gastroenterology, including workflow optimization and informed decision-making [7].

Despite encouraging initial experiences, several uncertainties remain regarding AI implementation in clinical practice. Key considerations for healthcare leadership include the logistics of implementation, associated costs, identification of end users, and the role of AI in operational efficiency [8]. Continued evaluation is necessary to fully realize the benefits of AI while navigating its accompanying challenges [8].

### 1.1. Background and Rationale

AI has emerged as a transformative tool in several specialties, especially gastroenterology, where it has the potential to optimize diagnosis, treatment, and patient care [9]. In modern gastroenterology practice, AI excels at managing and interpreting vast, complex datasets from diverse sources such as endoscopic and imaging devices, electronic health records, digital registries, governmental databases, social media, and wearable devices [10]. This ability supports a shift from traditional approaches toward a more personalized, predictive, and preventive paradigm in gastroenterology [10,11,12].

The adoption of AI-driven technologies using deep learning for image analysis in gastrointestinal endoscopy has occurred faster than anticipated [13]. In practice, AI has been associated with improved polyp detection during colonoscopies and increased diagnostic accuracy [9], and AI-supported systems for bowel preparation scoring, clinical reporting, and patient communication (including generative tools such as ChatGPT) are being explored [11,12,14].

Overall, AI offers tools that streamline workflows, support clinical decisions, and enhance patient engagement, directly addressing practical and organizational pressures in gastroenterology nursing. This technological shift is fostering a more efficient, accurate, and patient-centered care environment [11,12].

#### 1.1.1. Rationale for AI in Gastroenterology Nursing

AI in gastrointestinal endoscopy holds tremendous promise to enhance clinical performance, develop better treatment plans, and improve patient outcomes [15]. Gastroenterology involves managing large volumes of clinical data, including endoscopic images and patient records, making it well-suited for AI applications that analyze, interpret, and generate actionable insights [7,16]. Nurses in this specialty perform diverse roles ranging from pre-procedure assessment to chronic disease management, which require efficient documentation, rapid decision-making, and effective patient communication [16,17]. AI-powered tools—such as chatbots and automated documentation systems—can reduce the time nurses spend on routine administrative tasks, enabling them to devote more attention to direct patient care [17].

AI-based technologies such as machine learning, predictive analytics, and natural language processing are increasingly explored in gastrointestinal nursing as potential supports for more accurate, data-driven clinical decisions, patient monitoring, treatment planning, and workflow optimization. However, AI integration also raises important ethical and privacy considerations. It is essential that nurses actively engage in the design and implementation of AI systems to ensure these technologies complement rather than replace patient-centered care [18].

**Figure 1:**
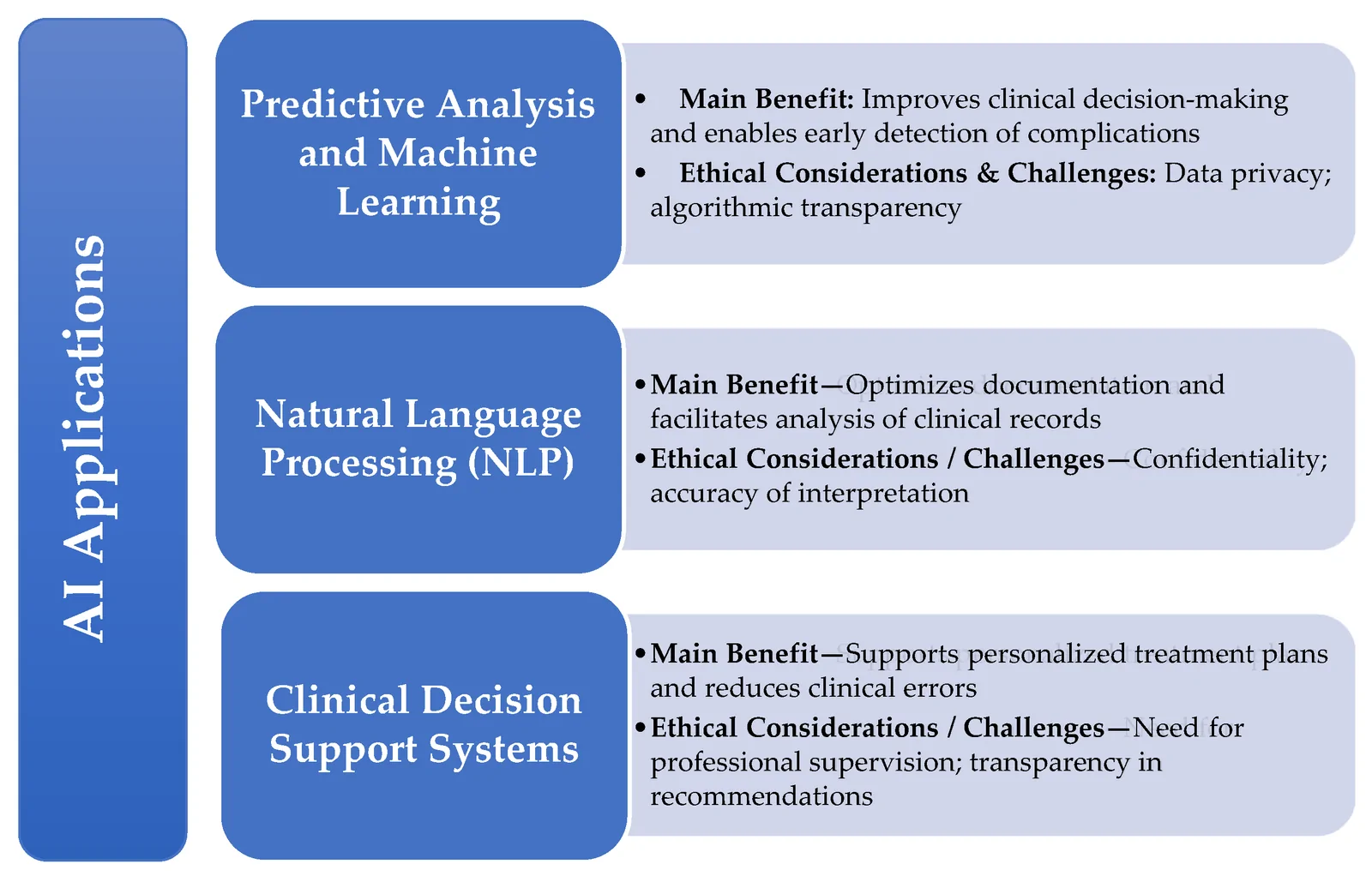
Main applications and challenges of AI in gastrointestinal nursing.

In sum, AI in gastroenterology nursing offers substantial potential to enhance care quality and operational effectiveness, but its success depends on addressing ethical challenges and maintaining the central role of skilled nursing judgment in clinical workflows (Figure 1).

#### 1.1.2. Opportunities and Early Evidence

Studies evaluating the use of chatbots in gastroenterology nursing have shown high satisfaction regarding in-hospital education and management, indicating that chatbots can effectively support patient education and clinical workflows during hospital stays [17]. However, there remains a critical need to optimize these tools for pre-hospital and post-discharge care to extend their benefits across the full patient care continuum.

AI models are increasingly applied for risk prediction, early disease detection, and personalized treatment planning in gastroenterology, serving as valuable aids to nurses managing complex clinical decisions [16,19]. These AI-driven supports allow for more precise and individualized patient care, improving monitoring and optimizing clinical workflows.

Chatbots, specifically large language models like ChatGPT, hold promise in improving patient–provider communication, providing tailored education, assisting in disease management, and even supporting research. Nonetheless, their current limitations include inconsistent responses, inability to fully grasp complex medical nuances, the need for ethical oversight, and ensuring they complement rather than replace professional nursing judgment (multiple studies reviewed in recent systematic analyses).

#### 1.1.3. Challenges

Despite the promising opportunities of AI in gastroenterology nursing, several key challenges remain. First, there are significant evidence gaps, with most research in early stages and a lack of nursing-specific, real-world studies to guide practical implementation [7,17]. This limits a clear understanding of AI’s impact and optimization for nursing workflows.

Secondly, bias and equity concerns pose major risks. AI systems, if not carefully designed and validated, can introduce or amplify existing health disparities by reflecting biases present in underlying data and healthcare practices [19]. Ensuring fairness in AI applications requires diverse data inclusion and ongoing evaluation.

Third, interpretability of AI tools is critical. Nurses require AI systems that provide transparent and understandable outputs to confidently integrate them into clinical decision-making and maintain safety and efficacy in patient care [7,16]. Opaque “black box” AI models hinder trust and appropriate use.

**Figure 2:**
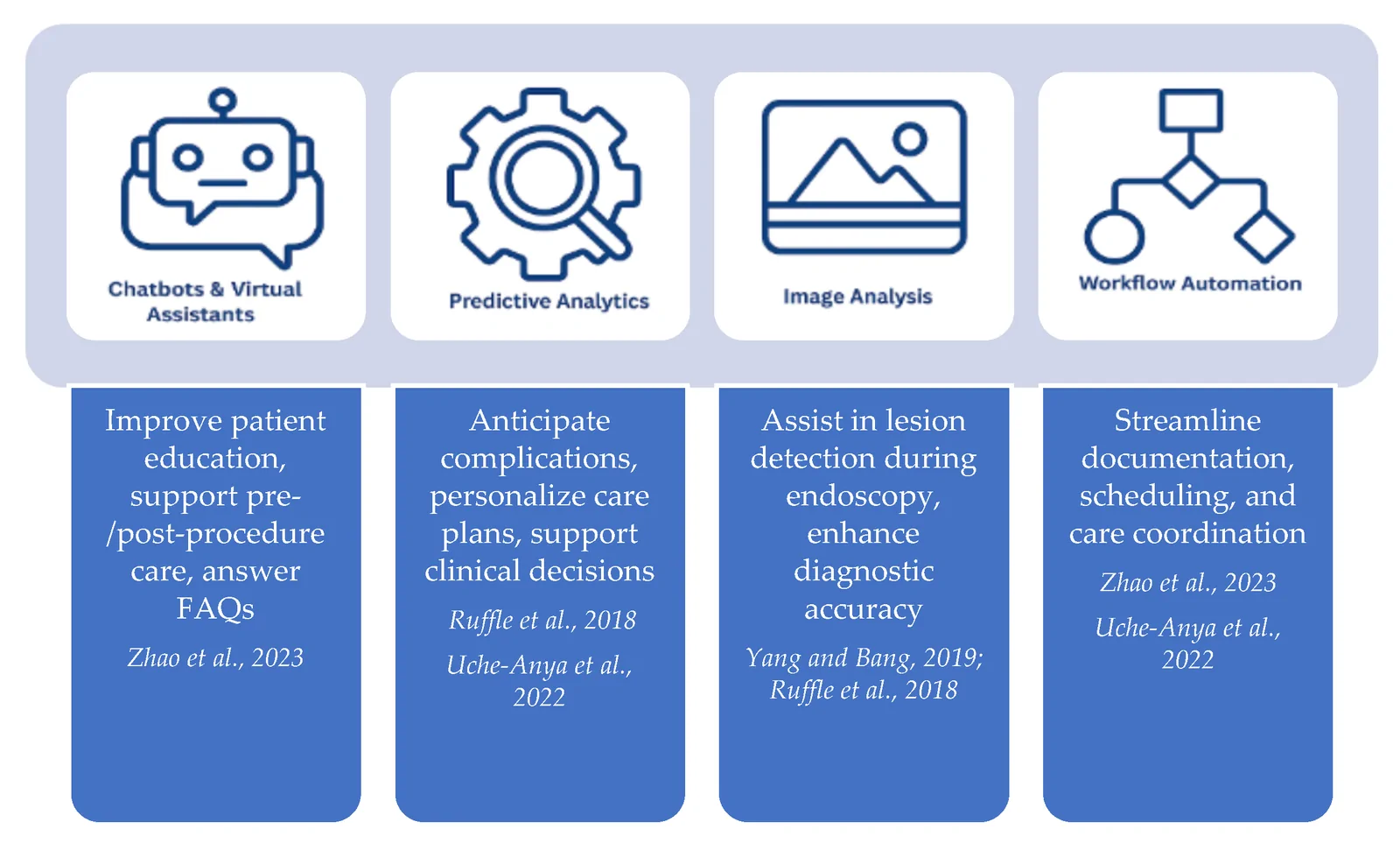
Potential Benefits for Nursing Practice per AI Application Area. Sources shown in the figure: Yang and Bang, 2019 [7]; Ruffle et al., 2018 [16]; Zhao et al., 2023 [17]; Uche-Anya et al., 2022 [19].

While AI offers the potential to alleviate administrative burdens, enhance clinical decisions, and improve patient engagement in gastroenterology nursing, it is imperative that targeted research, ethical design, and careful implementation address these challenges to fully realize AI’s benefits while safeguarding equity and usability (Figure 2).

### 1.2. Relevance of Nursing in Gastroenterology

Nurses play a central and multifaceted role in gastroenterology, contributing to endoscopy, IBD management, GI oncology, nutrition, and patient education. Their expertise is vital for high-quality, patient-centered care, especially as the complexity of gastroenterological conditions and treatments increases.

#### 1.2.1. Key Roles of Nurses in Gastroenterology

Gastroenterology nurses play vital roles across various clinical domains, providing advanced, specialized care that significantly impacts patient outcomes.

In endoscopy settings, nurses are instrumental from pre-procedure assessment through post-procedure recovery. Their responsibilities encompass preparing patients for procedures, monitoring safety during sedation, managing immediate aftercare, and delivering patient education to promote understanding and adherence. Nurses with specialized expertise in inflammatory bowel disease (IBD) related endoscopy tailor procedures to patients’ complex needs, optimizing both safety and clinical outcomes [20,21,22].

In IBD management, nursing practice extends to comprehensive therapeutic oversight, including coordinating and monitoring biologic therapies, ongoing patient education, and providing emotional support. Nurses act as liaisons to coordinate multidisciplinary care and ensure continuity through careful follow-up. Evidence shows that nurse involvement in IBD care improves patients’ quality of life, reduces hospital admissions, and increases satisfaction with care [22,23].

Within gastrointestinal oncology, nurses serve as essential members of the multidisciplinary team by managing symptoms such as pain and nausea, assessing and delivering nutritional interventions, and addressing psychosocial needs. Their holistic approach assists patients in navigating the challenges of cancer diagnosis, treatment, and survivorship [22,24].

Nutrition management is another critical nursing contribution, especially for patients with IBD or recovering from gastrointestinal surgery. Nurses assess nutritional status, manage or supervise enteral feeding, and educate patients on dietary strategies. Studies indicate that comprehensive nursing care combined with home enteral nutrition support improves clinical outcomes and enhances patient independence [22,25].

Patient education is a cross-cutting nurses’ responsibility underpinning all aspects of gastroenterology care. Nurses provide disease-specific information, promote treatment adherence, teach self-management skills, and act as important communication bridges between patients and the healthcare team. This proactive education empowers patients and contributes to better self-care and health outcomes [22,23,26].

**Figure 3:**
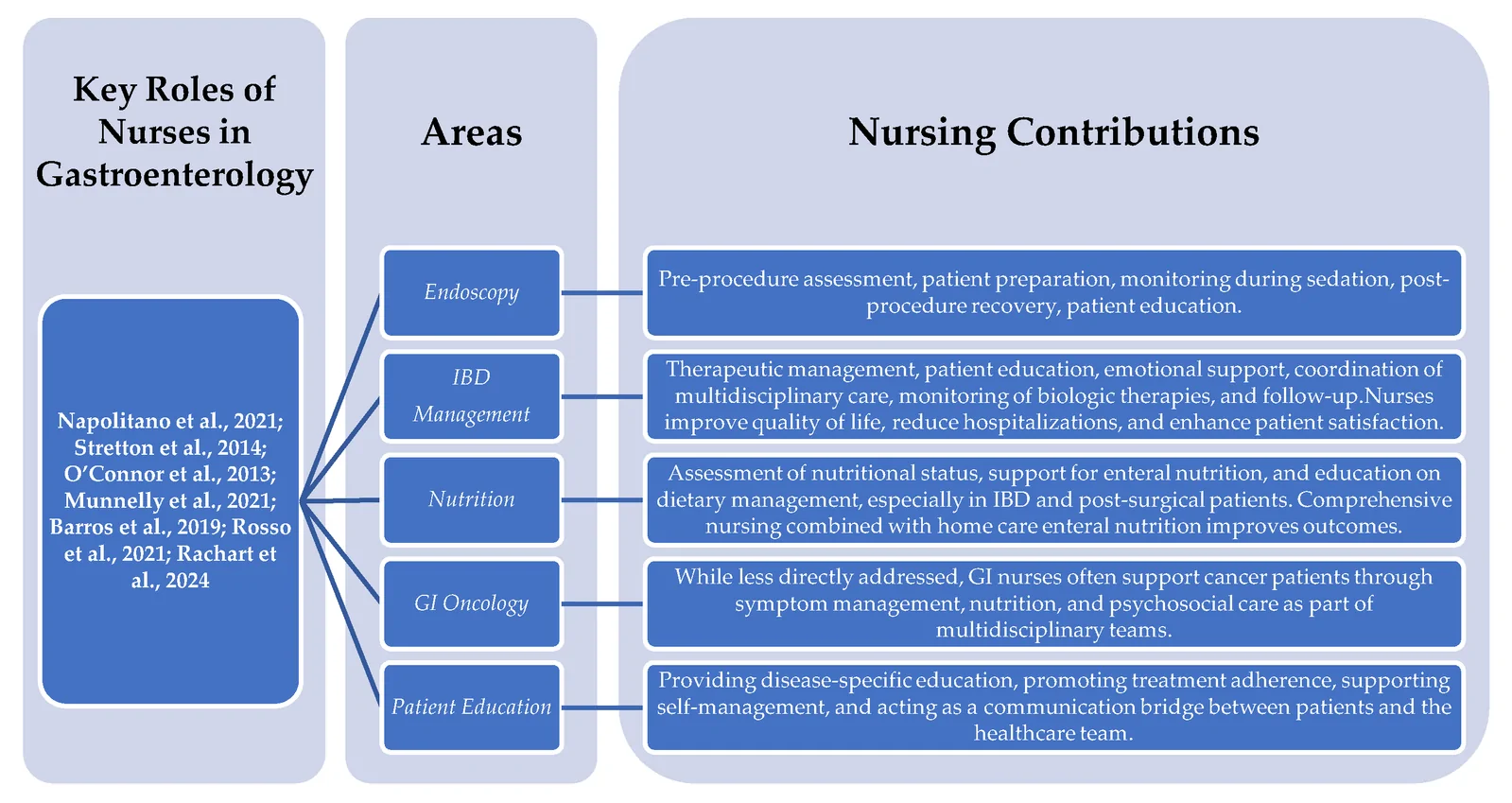
Key Roles of Nurses in Gastroenterology and Nursing Contributions per Area. Sources shown in the figure: Napolitano et al., 2021 [20]; Stretton et al., 2014 [21]; O’Connor et al., 2013 [22]; Rosso et al., 2021 [23]; Munnelly et al., 2021 [24]; Barros et al., 2019 [25]; Rachart et al., 2024 [26].

Across all these domains, evidence consistently supports that the diverse and skilled contributions of gastroenterology nurses are essential for quality care and measurably improve patient outcomes and healthcare experiences (Figure 3).

#### 1.2.2. Impact and Challenges

Nursing in gastroenterology has a significant and multi-dimensional impact but faces important challenges as clinical demands and complexity increase.

Gastroenterology nurses play a critical role in improving patient outcomes, quality of care, and patient satisfaction. Studies consistently demonstrate that when specialized nursing care is provided, whether through advanced bedside and “detail” nursing, comprehensive clinical guidelines, or multidisciplinary collaboration, patients experience reduced pain, fewer complications and adverse events, shorter hospital stays, and a substantial increase in satisfaction and understanding of their conditions [27].

Specialized nurses, particularly in areas such as IBD and endoscopy, provide pre-procedure assessment, sedation monitoring, patient and family education, and post-procedure support—all of which are linked to higher care quality and better long-term health outcomes. Nurses also play a vital role in nutrition assessment and management and in providing psychosocial support, especially for cancer and chronic disease patients; these interventions further improve patients’ quality of life. Effective communication and care coordination by gastroenterology nurses are frequently cited as directly contributing to improved clinical results and the efficient operation of multidisciplinary teams.

Despite these clear positive impacts, significant challenges remain. There are gaps in specialized education and advanced training, particularly for emerging therapies and subspecialty areas such as pediatric gastroenterology and IBD-focused endoscopy [7,20,24]. Ongoing professional development and structured competency frameworks are needed to address evolving patient complexity and the pace of technological innovation. The changing landscape of healthcare, including more complex patient populations, new therapies, and greater use of digital health, demands that nurses possess advanced clinical, technical, and communication skills. Inadequate staffing, high workload, and the need to consistently demonstrate advanced competencies in increasingly sophisticated roles can strain both individual nurses and teams.

Furthermore, effective integration and recognition of the gastroenterology nurse within multidisciplinary teams is essential for ensuring optimal care but can be impeded by role ambiguity or institutional limitations. Addressing these challenges requires investment in specialized nursing education, institutional support for advanced practice roles, and the fostering of environments where nurses are recognized as central to team-based, patient-centered gastroenterology care.

#### 1.2.3. Key Points

- **Quality of Care**: Specialized gastroenterology nurses are linked to improved patient outcomes, reduced hospital visits, and higher patient satisfaction [26,28,29];
- **Education and Training**: There are gaps in advanced competencies and specific knowledge (pediatric care, endoscopy in IBD), highlighting the need for ongoing education and competency frameworks [7,20,24];
- **Multidisciplinary Collaboration**: Nurses are key members of multidisciplinary teams, coordinating care and advocating for patients across settings [7].

**Figure 4:**
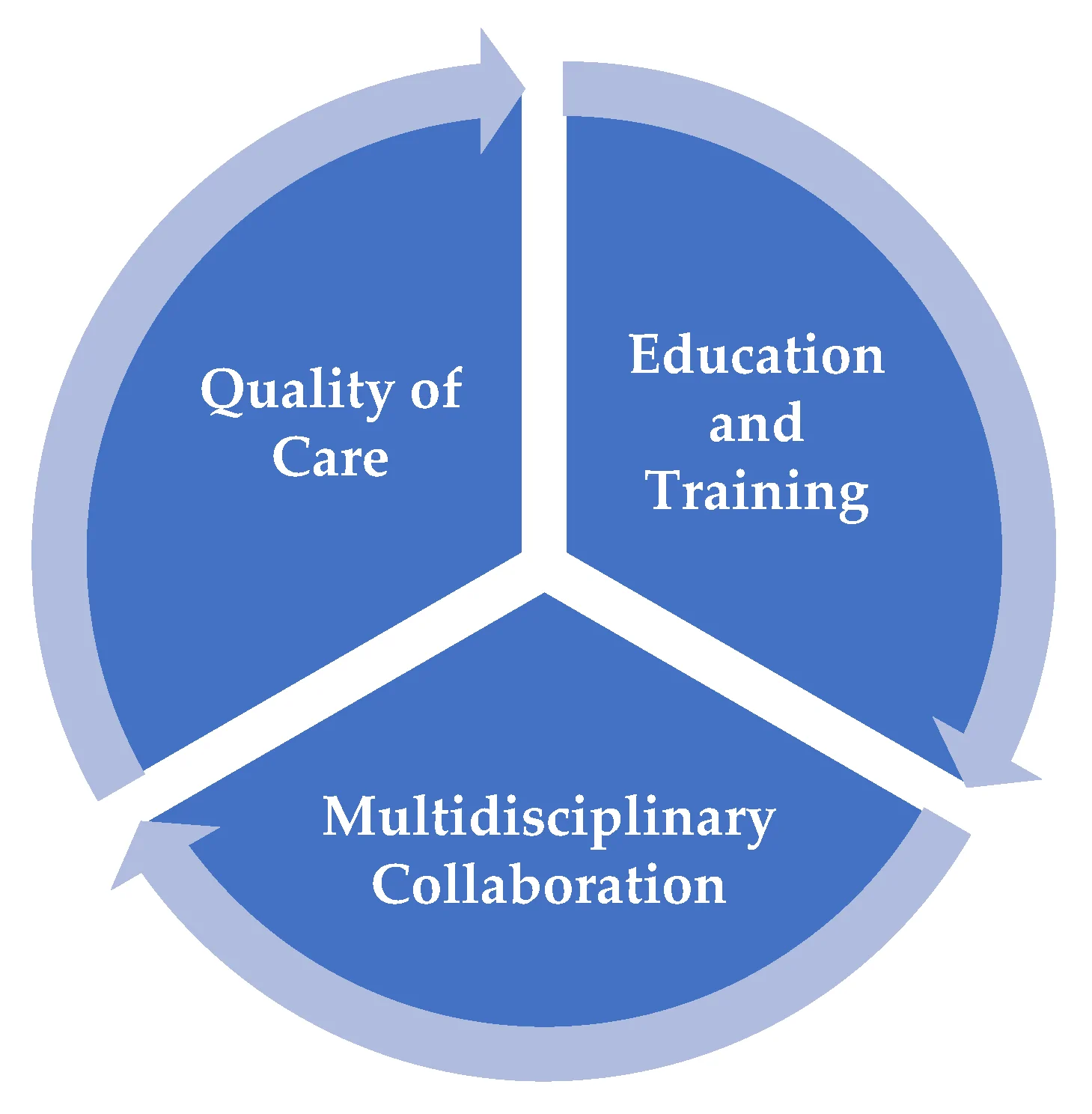
Impact and Challenges in Gastroenterological Nurse Care.

Nurses are indispensable in gastroenterology, providing expert care in endoscopy, IBD, nutrition, and patient education. Expanding specialized training and supporting their roles within multidisciplinary teams are essential for meeting the growing demands and complexity of gastroenterological care (Figure 4).

### 1.3. Justification for the Review

There is a clear need for a focused review on the intersection of AI, nursing, and gastroenterology, as the current literature primarily addresses AI’s impact from a physician or technical perspective, with limited attention to nursing roles.

#### 1.3.1. Gaps in the Existing Literature

- **AI in Gastroenterology**: Numerous reviews detail AI’s applications in areas such as disease prediction, endoscopic image analysis, and personalized medicine. However, these works largely focus on physician workflows and clinical outcomes, rather than on nursing practice [7,11,30,31].
- **AI in Nursing**: Some research explores AI integration in nursing—highlighting opportunities in decision support, patient monitoring, and workflow automation—but rarely addresses the unique context of gastroenterology nursing [1].
- **Lack of Intersectional Focus**: No comprehensive reviews specifically examine how AI tools are being adopted, adapted, or evaluated within gastroenterology nursing, nor do they address implications for nursing education, workflow, or patient care in this specialty [1].

#### 1.3.2. Advancements from the Review

- **Specialized Nursing Roles**: Gastroenterology nurses have distinct responsibilities in endoscopy, IBD management, oncology, and patient education—all areas that AI could transform. Yet, the literature does not systematically address these intersections [1].
- **Emerging Technologies**: As AI technologies become more prevalent in gastroenterology, understanding their impact on nursing roles, competencies, and patient outcomes is essential for safe, effective integration [1].
- **Guidance for Practice and Research**: A targeted review will identify best practices, evidence gaps, and future research priorities, supporting both clinical innovation and workforce development [1].

Despite the proliferation of research on AI in gastroenterology and in nursing, there remains a lack of intersectional reviews that specifically address how AI is being integrated into gastroenterology nursing—including its practical implications, required competencies, and patient care impacts. Addressing these gaps will directly support clinical advancement and professional development in this rapidly evolving field. To our knowledge, no prior review has specifically synthesized evidence at the intersection of AI, nursing practice, and gastroenterology using a structured scoping methodology; existing reviews address AI in gastroenterology broadly [7,11,30,31] or AI in nursing broadly [1,8,32], but not their combination. This review addresses that gap. Because a dedicated systematic (review-of-reviews) search for competing syntheses was not conducted, we describe this area as under-synthesized rather than asserting definitively that no such review exists; no review with this specific combined focus was identified in the course of conducting the present search.

### 1.4. Objectives

The primary objective of this scoping review is to identify and map the existing literature on AI applications relevant to nursing practice in gastroenterology. Specifically, the review aims to:

- Characterize the types of AI and AI-adjacent digital technologies used in gastroenterology settings with nursing involvement.
- Describe the clinical contexts in which AI is applied.
- Identify reported outcomes related to nursing practice, including workflow efficiency, decision support, and patient care.
- Examine challenges, limitations, and ethical considerations related to AI adoption in gastroenterology nursing.
- Highlight gaps in the literature and propose directions for future research, education, and policy development.

This review will provide a comprehensive overview and synthesis of how artificial intelligence is shaping nursing practice in gastroenterology, clarify areas needing further research or guidance, and inform both clinical practice and future educational initiatives in the field.

## 2. Methods

A comprehensive literature search was conducted to identify English-language publications relevant to AI, nursing, and gastroenterology. Major academic databases—including PubMed, Medline, Web of Science, and Scopus—were searched using broad keywords such as “artificial intelligence,” “nursing,” and “gastroenterology.” This strategy was designed to capture a wide spectrum of interdisciplinary research and ensure inclusivity of nursing-centric, gastroenterology-focused, and technology-driven studies published from 2000 onward.

The review utilized a narrative synthesis approach, appropriate for summarizing diverse study types, methodologies, and outcomes within a complex, emerging area.

### 2.1. Research Question

The guiding question for this scoping review was: “What is known about the use of artificial intelligence in nursing practice within gastroenterology?”

This inclusive research question allowed mapping of all the relevant literature where nursing intersects with AI within gastroenterology.

### 2.2. Eligibility Criteria

For the purposes of this review, artificial intelligence (AI) was operationally defined as any computational technology that performs tasks associated with human cognition, including machine learning, deep learning, natural language processing (NLP), large language models (LLMs) and generative AI, computer vision, and predictive analytics, applied to a clinical, administrative, or educational task. The review’s scope was deliberately defined more broadly than genuine AI alone: conventional rule-based clinical decision-support systems, digital or mobile health applications without a learning or predictive component, and standard automation (e.g., static reminder systems) were treated as AI-adjacent digital technologies and were eligible for inclusion, on an equal footing with genuine AI applications, wherever the technology was directly relevant to gastroenterology nursing workflows; this is reflected in the title, abstract, and objectives of this review. Each included study’s status against the strict AI definition above (met, partially met, or not met) is reported transparently in Section 3.2 (e.g., Marras et al. [33] and Manzari et al. [34], both of which explicitly report the absence of advanced AI, and were retained as AI-adjacent). The general topical eligibility criteria (studies at the intersection of AI/AI-adjacent digital technology, nursing, and gastroenterology) were defined before screening began and were not modified during the review process (Section 2.4); the specific strict/partial/not-met AI labels and the four-level nursing-involvement classification reported in Section 3.2 were formalized during revision as a transparency device to make explicit a distinction that was implicit, but not separately tabulated, during original screening.

The criteria for inclusion were studies that explicitly examined the intersection of artificial intelligence, nursing practice and gastroenterology, and were based on empirical studies, literature reviews and implementation or quality improvement reports. Only peer-reviewed articles published in English since 2000 were eligible, reflecting technological developments over the last two decades. Exclusion criteria included studies with a non-human/animal focus; publications focused only on the technical development of AI or validation of algorithms without clinical application or nursing; and non-peer-reviewed materials, such as dissertations, editorials without original data, or conference summaries. Titles and abstracts were screened for relevance against the inclusion and exclusion criteria independently and in duplicate by two reviewers, with disagreements resolved through discussion, and full texts of potentially eligible records were retrieved for further assessment. The results were organized thematically around study objectives, AI applications, clinical contexts, stakeholder functions and reported results. Special attention was given to the identification of evidence gaps, ethical concerns and implications for future practices and nursing education. Although the eligibility criteria as originally specified permitted literature reviews and implementation/quality-improvement reports alongside empirical studies, all 11 studies ultimately retained in the evidence synthesis (Section 3.1) are primary empirical or implementation studies; no secondary literature reviews were retained as included evidence, avoiding duplication between the evidence base and background citations. To characterize the strength of nursing relevance across included studies, each study was further classified by degree of nursing involvement using four levels: Level 1, nurses as primary users of the AI tool; Level 2, nurses as intervention providers or direct end-users; Level 3, nurses indirectly affected by the AI application (e.g., through changes to physician or interdisciplinary workflows); and Level 4, nursing implications inferred by the review team but not empirically evaluated in the primary study. This classification, together with an explicit check against the AI, gastroenterology, and nursing eligibility criteria for each study, is reported in Section 3.2.

### 2.3. Search Strategy

A comprehensive research strategy was developed in collaboration with a medical librarian to ensure complete coverage and relevance. The following electronic databases were searched: PubMed, Scopus, Medline and Web of Science. The complete per-database search strings, including controlled-vocabulary/MeSH terms, keyword synonyms, Boolean operators, truncation, date and language limits, and the date of the final search, are provided in Supplementary File S3 to support reproducibility.

The following information was extracted from each study: authors, year of publication and country, used AI method or technology, clinical scenario, nursing function, reported results, and study limitations.

The data were summarized quantitatively with descriptive statistics for categorical variables (types of AI technologies and clinical configurations) and qualitatively using thematic analysis to narratively summarize AI applications, nursing involvement and reported impacts. This combined approach has facilitated a comprehensive understanding of the current scenario of the literature regarding AI integration in gastroenterology nursing practice.

### 2.4. Protocol Registration and Reporting Standards

This scoping review followed the Preferred Reporting Items for Systematic Reviews and Meta-Analyses extension for Scoping Reviews (PRISMA-ScR) reporting guideline [35], and the completed PRISMA-ScR checklist is provided as Supplementary File S1. No protocol for this scoping review was registered in advance (e.g., on OSF or a similar registry). The eligibility criteria and search strategy reported in Section 2.2 and Section 2.3 were defined before screening began and were not modified during the review process. The classification scheme (strict-AI status and nursing-involvement level per study) was developed during revision to make this pre-existing scope explicit and auditable; it did not change which studies qualified for inclusion (Section 2.2).

No formal critical appraisal or risk-of-bias assessment of the included studies was performed. Consistent with JBI scoping-review guidance, which treats critical appraisal as optional for this study design, study-level limitations are instead reported narratively for each source in Section 3.1.

## 3. Results

The reviewed studies demonstrate a diverse range of AI applications within gastroenterology that hold promising implications for nursing practice. AI technologies such as convolutional neural networks for image analysis, machine learning models for risk prediction, natural language processing-based chatbots, and clinical decision support systems are increasingly integrated into clinical workflows with the potential to support and enhance nursing roles.

### 3.1. Search Results and Study Selection

**Figure 5:**
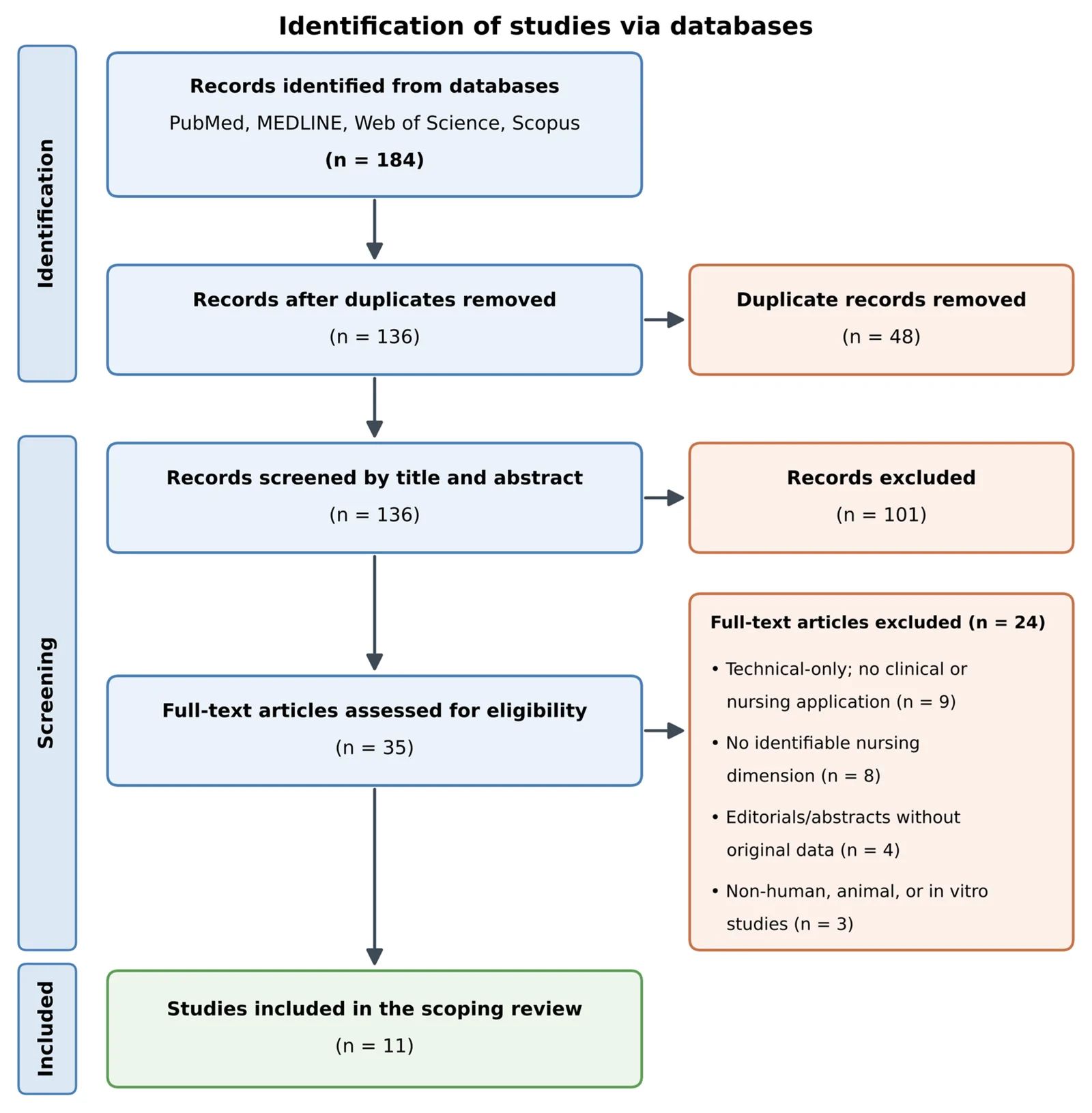
PRISMA-ScR flow diagram of database searching and study selection. Blue boxes show the identification, screening, and eligibility stages; orange boxes show the records removed or excluded at each stage (with reasons for full-text exclusion); the green box shows the studies included in the review. Arrows indicate the flow of records between stages.

Database searching across PubMed, Medline, Web of Science, and Scopus, together with screening against the eligibility criteria in Section 2.2, ultimately yielded 11 studies for inclusion in this review (Table 1). Database searching yielded 184 records; after removal of 48 duplicates, 136 unique records were screened by title/abstract, of which 101 were excluded. The remaining 35 full-text articles were assessed for eligibility against the criteria in Section 2.2, and 24 were excluded (9 technical-only, with no clinical or nursing application; 8 with no identifiable nursing dimension; 4 editorials/abstracts without original data; and 3 non-human/animal or in-vitro studies), leaving 11 studies for inclusion (Table 1; Figure 5).

As a reproducibility check on the identification stage of this search, PubMed’s public E-utilities interface was queried live on 14 August 2026 using the terms described in Section 2.3 (“artificial intelligence,” “nursing,” “gastroenterology,” English language, 2000–present; full query string and retrieved record list in Supplementary File S2), returning 33 records. Two of the 11 studies included in this review—Garcia et al. (2024) [36] and Vanalli et al. (2023) [37]—were independently recovered by this single-database re-run, providing partial, dated corroboration of the identification stage. Scopus and Web of Science, both used in the original search, require institutional subscription access that was not available for this check and could not be reproduced here; Medline is indexed within PubMed and was therefore not queried as a separate source. Figure 5 presents the study selection process as a PRISMA-ScR flow diagram. One further record identified during the search and cited elsewhere in this manuscript for background purposes—a 2025 multicentre cross-sectional survey modelling predictors of AI use among gastroenterology nurses [6]—was not carried forward as one of the 11 included studies because it evaluates nurses’ behavioural intentions toward future AI adoption rather than an implemented AI application in clinical use, and therefore does not meet the eligibility requirement that an AI technology be clearly identified and applied (Section 2.2); it is instead cited as contextual background in Section 4.2.

**Table 1:** Characterization of Included Studies.

Author, Year	Country	AI Method	Setting	Nursing Role	Outcomes	Limitations
Wang, Y.; et al., 2022 [38]	Taiwan	Utilized a U-Net convolutional neural network architecture for automated segmentation of fecal residue in colonoscopy images. The model was trained and validated on over 10,000 images extracted from 119 colonoscopy videos, manually annotated by experts to identify fecal residue. The model automatically segmented the bowel preparation quality by marking fecal-material-coated mucosa. Segmentation performance was assessed by area overlap metrics compared to manual annotation.	Clinical endoscopy center setting in Taipei Veterans General Hospital, Taiwan. Colonoscopy videos recorded with high-definition colonoscopes in real clinical practice.	Nurses could utilize the objective scoring tool for standardizing bowel preparation quality assessments and improving workflow and patient care related to colonoscopy preparation.	The model processed images rapidly (about 0.36 s per image). Achieved high accuracy: 94.7% ± 0.67% overlap area with manual segmentations. Strong correlation (r = 0.915, *p* < 0.001) between AI-predicted and manual measurement of fecal residue areas. The system can support real-time, quantitative and qualitative evaluation during procedures.	The model focused on segmentation of fecal residue, not a full clinical diagnostic AI system. Deployment and validation outside a single hospital setting remain to be verified. The nursing role was not directly explored or integrated into the study design.
Gu, F.; et al., 2024 [39]	China	Multiple machine learning models were used, including decision trees, support vector machines, extreme gradient boosting, and bidirectional projection networks, to predict inadequate bowel preparation. Decision tree model showed the best predictive capacity, with an area under the curve (AUC) of 0.80 in the validation cohort. The model inputs included patient characteristics, comorbidities, medication use, and bowel preparation quality.	Outpatient colonoscopy centers across multiple hospitals in China.	Not explicitly reported by the study; plausible involvement includes bowel preparation education, reinforcement, and protocol adherence, given the model’s predictive risk stratification. The model can inform nursing interventions tailored to patients at higher risk for inadequate bowel preparation.	The decision tree model predicted inadequate bowel preparation with an AUC of 0.80 in validation. Identified key risk factors, including body mass index, education level, use of simethicone, diabetes, age, previous history of inadequate bowel prep, and interval length. The model supports early identification to optimize bowel preparation quality, thus enhancing colonoscopy effectiveness.	The study focused on Chinese adult outpatients; applicability to other populations and inpatient settings requires validation. The nursing role and implementation of the model in clinical workflows were not detailed. External validation outside the participating centers is needed for broader generalization.
Fu, Z.; et al., 2024 [40]	China	The study developed Endo-chat, a medical chat application based on a large language model (LLM) combined with retrieval-augmented generation (RAG) technology. RAG integrates external department-specific knowledge bases and clinical guidelines (17 gastroenterology guidelines) to enhance answer accuracy and personalization compared to standard LLMs like ChatGPT.	Digestive endoscopy clinical nursing environments in hospital departments in Guangzhou, China	Nurses are end-users who benefit from the tool as it reduces their workload by automating answers to frequent patient questions related to digestive endoscopy care (preoperative, postoperative, diet, follow-up, etc.). Nurses gave positive feedback, perceiving the AI tool as a supportive assistant that improves efficiency.	Endo-chat outperformed traditional manual methods in response efficiency, completeness, and patient satisfaction. There was no significant difference in accuracy between Endo-chat and ChatGPT after knowledge base integration. Patients preferred AI services and supported AI integration in clinical nursing. Nurses reported that AI introduction reduced their communication workload.	Potential for some errors or incomplete information inherent in LLM technology, although reduced by RAG integration. Study restricted to digestive endoscopy nursing in specific Chinese clinical settings; broader applicability remains to be tested. Further work needed to address the low tolerance for errors in medical AI applications.
Zhao, Y.; et al., 2023 [17]	China	The study focuses on chatbots—AI-driven conversational agents—designed for use in gastroenterology nursing. Chatbots use natural language processing (NLP) to interact with patients and healthcare providers, delivering patient education, answering common questions, and supporting clinical workflows.	Gastroenterology clinical and nursing environments, where patient education and communication are essential.	Nurses use chatbots as supportive digital tools to enhance patient communication, education, and post-procedural care. Chatbots help reduce nurses’ workload by automating routine patient inquiries related to preparation, procedure, diet, and follow-up management. The technology augments nurses’ roles by improving efficiency and standardizing information delivery.	Chatbots improve patient understanding and satisfaction by providing timely, accurate information. Reduce nurse workload by automating frequently asked questions. Enhance standardization of education and improve nursing workflow efficiency. Promote patient engagement and adherence to care instructions.	Early-stage research with limited large-scale clinical validation. Chatbot effectiveness depends on quality of AI training data and language model capabilities. May not fully replace personalized nurse–patient interactions, especially for complex or emotional concerns. Implementation challenges include integration into clinical workflows and acceptance by staff and patients.
Garcia, P.; et al., 2024 [36]	United States	AI Method: Use of a generative artificial intelligence language model integrated within electronic health record (EHR) systems to draft initial physician reply messages to patients. Physicians then review and personalize these drafts before sending. The AI drafts empathetic, detailed message responses, which physicians edit to maintain personalized, high-quality communication.	Outpatient primary care and gastroenterology and hepatology clinics within an academic healthcare system (162 primary care and gastroenterology clinicians participated), where patient messaging through the electronic portal is routine.	Clinic nurses, alongside physicians and advanced practice clinicians, were among the care-team members who used the AI drafting tool to respond to patient portal messages, reflecting a team-based in-basket messaging workflow rather than a physician-only one. Nurses directly used AI-generated drafts to compose replies to patient messages, potentially reducing composition time and cognitive burden for nursing as well as physician and advanced-practice staff.	AI-generated drafts led to longer, more detailed physician responses, without reducing response time. Physicians reported that AI drafting eased the cognitive burden of composing messages, helping to overcome “writer’s block.” Patient satisfaction with message quality was maintained or improved due to more empathetic, thorough communication. Transparency was maintained by notifying patients that messages were AI-drafted but reviewed by their physician.	The study evaluated the drafting system in a limited set of clinical environments; generalizability to other settings requires further research. Physicians retained ultimate responsibility for message accuracy and personalization; risk remains if edits are insufficient. Reported quantitative outcomes (response length, response time, satisfaction) were pooled across physicians, advanced practice clinicians, and nurses and were not broken out by clinician type, so effects specific to nursing staff cannot be isolated.
Wu, L.; et al., 2023 [41]	China	The study developed a surveillance system incorporating clinical guidelines and patient data, with decision support logic embedded to manage follow-up after colorectal polypectomy. The system combines rule-based/regular-expression modules with a deep-learning-based text-to-structure information-extraction (UIE) model and natural language processing (NLP) to identify eligible patients and extract data from endoscopic/pathological reports—a hybrid design rather than a purely rule-based one. The system automated follow-up recommendations based on polyp pathology, patient risk factors, and clinical guidelines.	Multi-center clinical settings in China, involving hospitals performing colorectal polypectomies. Real-world outpatient clinical follow-up environments where surveillance scheduling and adherence were managed.	Nurses were responsible for manually informing patients of their surveillance recommendations before this system; the system automated that notification task, directly reducing nurse time previously spent on it (see Outcomes). Nursing staff benefited from the tool to improve patient management and workflow efficiency.	The system improved adherence to guideline-based follow-ups after polypectomy. Reduced delays in surveillance scheduling and enhanced the accuracy of follow-up timing compared to standard practice. Improved workflow efficiency for clinical staff managing post-polypectomy care. In a prospective trial, nurse time spent manually informing patients fell from 2.86 h (for 105 patients) to 0 h with system assistance—a directly measured reduction in nursing workload. Positive user feedback indicating acceptability and usability in busy clinical settings.	The surveillance system evaluated mainly for colorectal polypectomy; generalizability to other colorectal procedures or settings is untested. Although one module uses deep learning/NLP for information extraction, the core surveillance logic remains guideline/rule-based and does not yet incorporate predictive risk modelling or patient behavioral data. Long-term clinical impact on colorectal cancer prevention outcomes is not yet established. Implementation limited to Chinese healthcare settings; external validation needed.
Marras, C.; et al., 2024 [33]	Spain	Gamified, scenario-based digital educational tool (not a machine-learning/AI system); development and formative evaluation reported, with further validation still ongoing; clinical settings	The validation and use of the tool took place in various clinical settings in Spain, involving real-world healthcare professionals from hospitals and outpatient care settings where biosimilars are prescribed and implemented.	Nurses were primary users and end beneficiaries of the tool. The tool is designed to improve their confidence and communication skills for supporting patients during transitions from originator biologics to biosimilars. Nursing professionals participated actively in the tool’s validation, and the training is directly relevant to clinical nursing practice during medication switches.	Improved communication skills among clinicians regarding biosimilar switching. Increased healthcare provider confidence and preparedness, particularly for nurses supporting patient transitions. Potential reduction in nocebo effect (negative patient responses due to expectations). Positive feedback on tool usability, satisfaction, and intent to integrate into practice.	The validation was limited to a pilot/sample cohort and mainly short-term user experiences. Broader generalizability is uncertain; further studies are needed to confirm the tool’s effectiveness in diverse settings and over longer timelines. Results may best reflect Spanish healthcare contexts and might not fully translate to other systems without adaptation.
Manzari, Z.S.; et al., 2024 [34]	Iran	Randomized clinical trial with three arms: teach-back (TB), smartphone application, and control. No advanced artificial intelligence; the intervention compared traditional teach-back education (interactive verbal method) to a self-guided smartphone application for self-care education.	Clinical setting, specifically patients with inflammatory bowel disease receiving care in Mashhad, Iran.	Nurses functioned as educators and interventionists. In the TB group, nurses provided education via individual virtual video sessions and follow-up support, integral to both delivering and structuring educational content.	Both TB and app interventions led to significantly improved adherence to treatment compared to routine education. Mean post-test adherence scores: TB group 170.04, app group 167.99, control 159.60 (*p* = 0.003 for group differences). No statistically significant difference between TB and app groups (*p* = 0.989). Both interventions recommended—smartphone app highlighted as more cost-effective and accessible.	Single-center, short-term trial; generalizability may be limited to similar clinical and cultural settings. Small sample size; longer-term effects and clinical outcomes (beyond adherence) not assessed. Did not blind participants or care providers.
Garg, S.K.; et al., 2023 [42]	United States	Developed an automated clinical decision support application embedded in electronic health records (EHR) to assist with sedation triage for routine colonoscopy patients. The system uses rule-based algorithms with patient clinical data extracted from the EHR (demographics, comorbidities, previous sedation history) to classify patients into sedation risk categories.	Outpatient endoscopy suites in one or more healthcare centers performing routine colonoscopies.	Nurses involved in operationalizing sedation triage decisions supported by the EHR tool. The application aids nursing staff in efficient pre-procedural assessment, patient monitoring, and sedation planning. Enhances safety by standardizing sedation risk assessment and streamlining communication among the clinical team.	The tool demonstrated high accuracy in predicting appropriate sedation needs consistent with expert clinical judgment and existing sedation protocols. Improved standardization of sedation triage decisions compared to manual methods. Reduced time required for pre-procedural evaluations. May contribute to improved patient safety and procedural efficiency.	Study conducted in limited clinical settings; generalizability requires validation in wider populations and healthcare systems. The AI method relies on EHR data quality and completeness, which may vary. Sedation decisions may still require clinician and nursing oversight beyond tool recommendations. No long-term outcomes or patient satisfaction data reported yet.
Vanalli, M.; et al., 2023 [37]	Italy	AI Method: Utilization of NLP to analyze unstructured free-text nursing notes and the SMASH method for cross-mapping these notes to standardized nursing activities using string similarity metrics like Levenshtein distance and Jaro–Winkler distance.	Inpatient units (Medicine, Neurology, Gastroenterology) at Agostino Gemelli IRCCS University Hospital Foundation, Rome, Italy.	Focused on capturing the actual nursing activities documented in routine clinical care through free-text notes and mapping them to standardized nursing terminologies, facilitating better data usage for clinical practice, research, and policy development. Supports nurses by enabling systematic extraction and comparison of nursing interventions through standardized documentation.	In total, 82.3% (451) of unstructured free-text nursing activities were successfully mapped to standardized nursing activities using this method, with an additional 8.7% partially mapped and 9% unmapped. The automated mapping achieved high reliability metrics: recall 0.95, precision 0.94, accuracy 0.91, and F-measure 0.96, indicating strong performance of the NLP and SMASH system. The study underscored that lexical similarities exist between unstructured nursing notes and standard terminologies, enabling effective cross-mapping.	Retrospective single-center study limited to specific hospital inpatient units, potentially affecting generalizability. Some nursing activities could not be mapped due to terminology variability or novel/uncommon terms. Method requires expert consensus in the mapping process, which may be resource-intensive.
Zhai, Y.; et al., 2024 [43]	China	AI techniques applied include machine learning algorithms (e.g., random forest, support vector machine, XGBoost) to analyze electronic health record (EHR) data and clinical variables. Large dataset of cirrhosis patients from multiple centers was used to train and test predictive models.	Setting included tertiary hospitals with access to detailed clinical and laboratory data.	Indirectly relevant for nursing staff involved in managing cirrhosis patients, as improved prognosis evaluation tools can support nursing care planning, risk stratification, patient education, and monitoring. AI prognosis tools may assist nurses in identifying high-risk patients who need intensified care or education.	AI models significantly outperformed traditional scoring systems in accuracy (e.g., higher AUROC values) for predicting 1- and 3-year survival probabilities and adverse event risk. The models identified novel prognostic factors and complex interactions among clinical variables. Demonstrated potential for clinical decision support to tailor management and improve outcomes.	Retrospective design with potential biases related to data quality and missing values. External validation needed in broader and prospective cohorts. AI model interpretability and integration into clinical workflows remain challenges. Nursing-specific outcomes or workflow impacts were not directly studied.

### 3.2. Characteristics of Included Studies

The eleven included studies represented a mix of study designs and methodologies, reflecting the exploratory nature of AI applications in gastroenterology nursing. The majority were observational studies [38,39,41], including both prospective and retrospective designs. Two studies [17,36] adopted quasi-experimental, or implementation-focused approaches. Geographically (Table 2), most studies originated from China (*n* = 5), followed by the United States (*n* = 2), Taiwan (*n* = 1), Spain (*n* = 1), Italy (*n* = 1), and Iran (*n* = 1), reflecting a global but uneven distribution of AI research in this domain. The eligibility-criteria check and nursing-involvement classification of each included study are presented in Table 3.

The included studies encompass a range of methodologies and settings reflecting the diversity of artificial intelligence (AI) applications in gastroenterology nursing. Most studies employ quantitative designs, including retrospective analyses and clinical trials, while a smaller subset incorporates qualitative feedback or mixed methods to assess AI tool usability and impact. Geographically, the studies span a broad range of countries including Taiwan, China, the USA, Italy, Spain, and Iran, highlighting global interest and variation in AI adoption across healthcare systems. AI methodologies are diverse, featuring convolutional neural networks (CNNs) for image segmentation (fecal residue detection in colonoscopy videos), multiple machine learning (ML) algorithms (decision trees, support vector machines) for risk prediction and prognosis in diseases like cirrhosis and inflammatory bowel disease (IBD), natural language processing (NLP) applied to nursing documentation and chatbots, retrieval-augmented generation techniques in clinical communication tools, and rule-based clinical decision support systems embedded within electronic health records.

Clinical settings primarily include endoscopy units, outpatient colonoscopy centers, inpatient wards specializing in gastroenterology care, and digital health environments supporting patient education and communication. Nurse roles captured in these studies range from operationalizing AI tools for workflow enhancement, such as bowel preparation scoring and sedation triage, to direct patient education, communication facilitation via chatbots, care coordination, and digital documentation standardization.

Outcomes evaluated commonly focus on AI accuracy, predictive performance (AUC values for classification tasks), workflow efficiency gains, reductions in nursing workload, patient satisfaction, and nursing confidence in clinical tasks. Several studies report high performance of AI models with accuracies often exceeding 85–90% and meaningful improvements in clinical processes. However, many note limitations including single-center designs, restricted population diversity, lack of long-term or external validation, and limited explicit assessment of nursing workflow integration and patient outcomes. Overall, the body of evidence illustrates promising technological advances with potential for significant clinical benefits in gastroenterology nursing. Nonetheless, it underscores the need for further research to validate these tools across broader settings, integrate nursing perspectives systematically, and address ethical, educational, and implementation challenges. To keep distinct levels of evidence from being conflated, this review distinguishes four outcome types across the included studies: technical/model outcomes (e.g., segmentation accuracy, AUC, precision/recall), workflow outcomes (e.g., time saved, standardization of a process), nursing outcomes (e.g., nurse workload, confidence, or task performance directly measured), and patient outcomes (e.g., adherence, satisfaction, safety). As Table 1 and Table 3 show, most included studies report technical or workflow-level outcomes only; nursing- or patient-level outcomes were directly measured in a minority of studies, and this distinction is maintained throughout Section 3.3, Section 3.4, Section 3.5 and Section 4.1.

### 3.3. AI Applications in Nursing Practice

Building on the overview in Section 3, this subsection details how each AI technology identified across the 11 included studies maps onto specific nursing-relevant applications, spanning decision support, workflow optimization, patient education, and endoscopy-related tasks.

The main benefits of the adoption of AI applications in Nursing Practice are as follows:

- **Enhanced Workflow Efficiency and Decision Support**: AI tools automate labor-intensive tasks such as bowel preparation quality assessment [38], patient risk stratification [39], and sedation triage [42]; direct nursing use and workflow benefit were reported for sedation triage [42], whereas for bowel-preparation image scoring [38] and risk-stratification modelling [39], nursing relevance was inferred by the review team rather than directly evaluated by the primary studies (Table 3). Where directly evaluated, such tools can improve workflow efficiency and enable nurses to focus more on direct patient care. Chatbots and conversational AI [17,40] can reduce communication burdens by automating routine patient inquiries, standardizing education, and supporting care coordination. This enhances nurse productivity and patient engagement.
- **Improved Clinical Outcomes through Predictive Modeling**: Machine learning models show strong potential in predicting patient risk factors and outcomes, such as inadequate bowel preparation or prognosis in cirrhosis [39,43]. These predictive insights can inform nursing care plans, enabling more personalized interventions and timely management.
- **Augmented Patient Education and Support**: AI and AI-adjacent digital tools, including chatbots and a digital simulation trainer [33], support nurses in educating patients about complex treatments, medication transitions, and self-care, leading to improved adherence and empowerment. Such tools complement nursing efforts to provide consistent, accessible information.
- **Standardization and Data Utilization**: Natural language processing and mapping tools [37] facilitate the standardization of nursing documentation, enabling better tracking of nursing activities and outcomes. This can enhance research, quality improvement initiatives, and policy development.

Despite these advancements, studies highlight limitations and raise careful considerations regarding AI’s integration into gastroenterology nursing:

- Most AI tools remain in early implementation stages or are validated within limited geographic or clinical settings, requiring broader external validation;
- AI applications rarely directly integrate nursing roles into their design or outcomes assessment;
- Ethical considerations, including transparency, error tolerance, data privacy, and equitable care, must be addressed as AI use grows.

There is a need for nurse involvement in AI development to ensure tools meet clinical needs and preserve the nurse–patient relationship.

#### 3.3.1. AI-Assisted Decision Support

Several studies described how AI could augment clinical decision-making in nursing practice. Gu et al. [39] developed several machine learning models (including decision trees, support vector machines, and XGBoost) to predict inadequate bowel preparation before colonoscopy, a critical determinant of procedural success; the decision-tree model performed best (Table 1). Though nurses were not direct model users, the tools supported nursing roles in pre-assessment and patient preparation.

Applications such as IBD flare prediction, nutrition risk scoring, and symptom triage were not evaluated in any of the 11 included studies and are therefore not reported as findings of this review; potential future directions of this kind are discussed in Section 4.6.2.

#### 3.3.2. Workflow Optimization

Workflow support was a prominent theme in studies involving large language models (LLMs). Fu et al. (2024) [40] developed Endo-chat, an LLM-based chat application with retrieval-augmented generation (RAG), to automate responses to patients’ routine questions in digestive endoscopy nursing, reducing the time nurses spend answering repetitive inquiries [40]. Similarly, Garcia et al. (2024) [36] evaluated AI-generated draft replies to patient inbox messages; although AI drafting did not significantly reduce physicians’ response time, it eased the cognitive burden of composing replies and was associated with more detailed, empathetic communication [36]. These tools indirectly benefit nursing by alleviating administrative burdens and enabling more time for direct patient care [36]. Wu et al. (2023) [41] also demonstrated how AI can assist with patient follow-up coordination post-polypectomy, a task often managed by nurses. Their AI-based surveillance system automatically tracked patient adherence and follow-up appointments, ensuring care continuity [41].

#### 3.3.3. AI in Patient Monitoring and Education

Chatbot applications were discussed by Zhao et al. [17], who implemented conversational agents to support patient education, answering common GI-related questions and facilitating ongoing communication. These AI tools were shown to reduce nursing workload while enhancing patient satisfaction. Although not a replacement for human interaction, such technologies offer scalable communication channels that support patient self-management [17]. Wearables and AI-integrated monitoring tools were not specifically addressed in any of the included studies; this potential future direction is discussed in Section 4.6.2 rather than reported here as a finding.

#### 3.3.4. AI in Endoscopy Nursing

Wang et al. (2022) [38] showcased the use of deep learning—specifically a U-Net convolutional neural network—to automatically segment fecal material in colonoscopy images. This supported objective evaluation of bowel cleanliness, a task often performed subjectively by nurses and physicians. While still in the research phase, such tools could standardize preparation quality assessments and reduce interobserver variability.

#### 3.3.5. Applications Not Evaluated in the Included Studies

Sedation monitoring, instrument tracking, and adverse-event prediction were not evaluated in any of the 11 included studies and are therefore not reported here as findings; they are discussed, together with the points raised in Section 3.3.1 and Section 3.3.3, as potential future directions in Section 4.6.2.

### 3.4. Impact on Nursing Practice

Across the studies, several positive impacts on nursing practice were reported or implied:

- Technical improvements in objective image-analysis accuracy that could support nursing-led bowel-preparation scoring, though nursing use of the tool was not directly evaluated by the primary study [38];
- Greater efficiency in documentation and communication [36,40];
- Enhanced patient interaction through chatbots allowing nurses to focus on higher-order tasks [17];
- Support for follow-up management, a traditionally labor-intensive task, via automated systems [41].

Despite these benefits, the ethical and legal dimensions of AI implementation were minimally addressed. Concerns included:

- Data privacy risks, especially in communication tools.
- Algorithmic bias and lack of transparency in decision support systems.
- Liability ambiguity, particularly when AI-generated outputs guide care.
- The preservation of the nurse–patient relationship, which could be threatened by over-reliance on automation.

### 3.5. Identified Gaps

Several gaps were consistently noted across the evidence base:

- **Limited nurse-led research**: Most studies were designed and executed by multidisciplinary or medically oriented teams, with nurses playing passive rather than active roles. There is a pressing need for nurse-led innovation and evaluation to ensure AI tools align with nursing values and workflows.
- **Poor external validation**: Many AI tools were validated only in single-country or single-center settings. Broader validation across diverse populations and clinical environments is necessary [38,39].
- **Lack of implementation-focused studies**: While the technical feasibility of AI tools is often demonstrated, real-world studies examining implementation processes, usability, workflow integration, and sustainability remain scarce.
- **Ethical oversight**: Few studies addressed regulatory frameworks, algorithmic accountability, or informed consent in AI-supported care, critical components for safe and equitable adoption.

## 4. Discussion

### 4.1. Summary of Main Findings

This review revealed a dynamic and evolving application of AI technologies across gastroenterology and gastroenterology nursing. The most common AI applications were concentrated in clinical decision support, image analysis for bowel preparation, documentation assistance, and patient communication. Studies such as those by Wang et al. [38] and Gu et al. [39] highlight AI’s ability to improve the quality and efficiency of colonoscopy preparation through predictive modeling and automated image segmentation [38,39]. Meanwhile, Fu et al. [40] and Garcia et al. [36] demonstrated the value of large language models (LLMs) in enhancing documentation workflows and managing patient inbox messages, core responsibilities within nursing practice [36,40]. Notably, studies illustrate how chatbots can reduce the communication burden on nursing staff while maintaining patient engagement [17]. Across these implementations, the included studies reported gains in workflow efficiency, technical/diagnostic accuracy, and reduced administrative load; evidence of more timely patient interaction was limited to a single study and did not reach statistical significance [36]. However, while nursing roles were often indirectly impacted, there were few studies led or conceptualized by nurses, indicating a gap in nursing-driven AI research.

### 4.2. Integration with Clinical Practice

The integration of AI into routine clinical practice remains uneven. Some systems, particularly predictive and communication-support tools, show promising alignment with existing workflows and electronic health records (EHRs), as evidenced by [36]. However, readiness across healthcare settings is variable and often dependent on institutional infrastructure and leadership support. The adoption of artificial intelligence requires a thorough understanding of its capabilities, limitations, and ethical implications, ensuring its use is safe, relevant, and aligned with patient needs [44]. There are concerns regarding the use of AI in clinical practice related to its technical aspects and the cost coverage associated with its implementation [45]. Nurses recognize that AI can improve clinical decision-making, optimize workflows and reduce errors, especially in areas such as risk forecasting and documentation [6]. The integration of digital health technologies into the healthcare sector has immense potential to increase efficiency, improve patient outcomes, and generate economic benefits. However, widespread adoption of these technologies has been hampered by a lack of digital skills among healthcare professionals. To close this digital skills gap, it is critical that healthcare education and training programs prioritize the development of digital proficiency [46]. For nurses, successful integration depends significantly on digital upskilling and workflow adaptation. Integrating AI into nursing offers opportunities to improve care but requires leadership and active participation from nurses to ensure that the technology complements, rather than replaces, compassionate and ethical care [32]. As AI tools become more embedded in clinical tasks—such as documentation, monitoring, and patient triage nurses will require not only technical competencies but also critical thinking skills to interpret and validate AI-generated outputs. The studies reviewed suggest that while AI can support care delivery, its adoption requires organizational readiness, appropriate training frameworks, and a culture of trust and transparency.

### 4.3. Challenges and Barriers

Despite its promising potential, the adoption of AI in gastroenterology nursing faces several significant challenges and barriers that must be thoughtfully addressed to ensure ethical, effective, and sustainable use.

A primary concern is data privacy and security, particularly because AI systems such as chatbots and surveillance tools process sensitive patient information. Adhering to stringent privacy regulations and enforcing institutional safeguards to protect patient data is essential and foundational for AI deployment [41]. Without robust protections, risks of data breaches and misuse could undermine patient trust and clinical safety.

Digital literacy presents another key barrier. Nurses require adequate training not only to operate AI tools but also to critically interpret, validate, and respond appropriately to AI outputs. This extends beyond traditional clinical education, necessitating new competencies and ongoing professional development. Resistance may arise if AI is perceived as replacing nurses’ clinical judgment rather than supporting it, emphasizing the need for clear communication about AI’s complementary role.

Regulatory frameworks lag behind AI innovations, creating gaps in accountability, liability, and ethical oversight. Many AI algorithms operate as “black boxes,” limiting transparency and interpretability. This opacity raises concerns about the trustworthiness of AI recommendations and complicates responsibility in adverse events. Regulatory bodies need to evolve standards that ensure explainability, safety, and fairness [6,30].

Moreover, implementation costs and logistical challenges contribute to significant barriers. Healthcare organizations must consider the financial burden of AI infrastructure, maintenance, and training. Defining who uses AI and how it fits into existing workflows also remains a practical dilemma that clinic leadership must address to optimize efficiency without disrupting care [47].

Further challenges include potential bias in AI models caused by unrepresentative training data, leading to inequitable care outcomes. AI’s limited capability in handling complex or nuanced clinical scenarios can restrict its utility, requiring well-defined limits of use and continuous evaluation [48].

Addressing these challenges demands structured implementation strategies that actively involve nurses, patients, and other stakeholders. Supervised clinical trials and real-world validation studies are crucial to assess AI effectiveness and safety on a large scale. Ethical frameworks must balance innovation with patient rights, equity, and clinician autonomy to ensure AI enhances rather than undermines quality care.

In summary, while AI holds transformative promise for gastroenterology nursing, overcoming barriers related to privacy, literacy, regulation, cost, and bias is essential to responsibly harnessing its benefits for clinical practice and patient outcomes.

### 4.4. Comparison with Other Specialties

Compared to other specialties such as oncology and cardiology, AI integration in gastroenterology nursing remains emergent and less mature. In oncology nursing, AI is already being leveraged meaningfully in clinical decision support for chemotherapy management, symptom prediction, and longitudinal patient-reported outcome tracking, allowing nurses to play a central role in AI-assisted care coordination and triage. Cardiology nursing similarly benefits from AI tools used for electrocardiogram (ECG) interpretation, heart failure risk stratification, and remote patient monitoring, which provide nurses with actionable insights and support for managing acute and chronic cardiac patients.

Gastroenterology’s unique procedural and preparatory care, including bowel preparation and endoscopic imaging, distinguishes it from these fields. AI applications such as convolutional neural networks for image segmentation (bowel prep quality) and predictive modeling for risk stratification are highly relevant. However, the adoption of AI in gastroenterology nursing is less integrated and less evaluated from the nursing perspective, in contrast to oncology and cardiology where nurse-driven AI tools are more established. This indicates an opportunity for gastroenterology nursing to build on lessons learned from the more mature implementations in other specialties, focusing on involving nurses directly in AI development, evaluation, and clinical workflow integration to realize broader benefits.

In summary, while oncology and cardiology showcase more advanced and nurse-centered AI applications, gastroenterology nursing is poised for growth in this area, with specific attention needed on tailoring AI tools to procedural workflows and patient education roles that define the specialty.

### 4.5. Implications for Nursing Education

AI is rapidly advancing and increasingly integrated across healthcare professions, including nursing. This shift creates both opportunities and challenges for nursing education to equip future nurses with the competencies needed for a digitally enabled healthcare environment. Academic institutions play a crucial role in training the next generation of nurses with skills spanning AI literacy, computational biology, data science, and bioinformatics through collaborative programs blending theory with real-world clinical experience [49]. Such training is vital to preparing nurses to use AI tools competently and ethically in practice. Structured educational frameworks that embed digital skills, including foundational AI concepts, ethical implications, and practical clinical applications, are essential. Integration of AI literacy into basic nursing curricula and continuing professional development will foster proficiency in AI-enabled workflows, enhance clinical decision-making, and enable personalized care delivery [46,50]. Continuing education should emphasize hands-on experience with AI tools through workshops, online courses, and simulation-based training programs. Simulation offers safe environments for nurses to familiarize themselves with AI-assisted decision support and documentation tools, such as large language models and chatbots, addressing nuances like language processing and interpretation [40,51]. Nursing curricula need to evolve to encompass ethical considerations, regulatory frameworks, and the social implications of AI, promoting responsible use and safeguarding patient rights [14,52]. Interprofessional education encouraging collaboration among nurses, data scientists, engineers, and physicians is also key to the successful development and evaluation of AI tools tailored to clinical needs.

Overall, AI integration demands a commitment to lifelong learning and continuous skill development among nurses, supported by scientific societies and institutional policies promoting AI literacy. This will ensure nurses remain proactive users and advocates for AI in gastroenterology and broader healthcare, ultimately improving patient outcomes and care experiences.

### 4.6. Research Implications

In the evolving landscape of digital health, artificial intelligence (AI) offers significant potential to transform nursing practice by enhancing diagnostic accuracy, optimizing care plans, and supporting resource allocation. However, its integration also raises important ethical concerns, notably around data privacy and algorithmic bias, necessitating comprehensive workforce training to ensure safe and effective use [53].

Building digital literacy among healthcare professionals will be a critical driver in achieving seamless collaboration between technology and care delivery, ultimately improving patient experiences and outcomes [46]. This review highlights several key research priorities for advancing AI in nursing: Interdisciplinary, Prospective, and Implementation-Focused Research.

Currently, most studies are preliminary proof-of-concept or retrospective analyses. There is a pressing need for large-scale, prospective, real-world evaluations that measure long-term impacts of AI on nursing workload, patient safety, care quality, and cost-effectiveness.

#### 4.6.1. Nurse-Led AI Research and Development

Nurses frequently appear as end-users or beneficiaries in studies but are rarely leading co-developers or evaluators of AI systems. Encouraging nurse-led research will ensure AI tools better align with the realities of clinical practice and uphold patient-centered values. Nurses should actively participate in design, testing, and policymaking processes to maintain ethical integrity and preserve humanistic care [32]. Nurses can play an instrumental role in the following areas:

- **Equity and Ethics**: Broad AI deployment demands sustained investigation into ethical issues such as bias mitigation, privacy protection, and informed consent. Understanding AI’s impact on nurse–patient relationships and performance across diverse populations is essential to foster equitable healthcare delivery [54].
- **Training and Institutional Support**: Successful AI integration requires investment in nurse education, institutional infrastructures, and clear regulatory guidelines that support transparency, quality assurance, and accountability in AI use [6,12].
- **Collaboration Between Developers and Practitioners**: Fostering partnerships between AI developers and frontline practitioners will promote the creation of clinically relevant, usable AI solutions tailored to gastroenterology nursing needs, thereby enhancing effectiveness and uptake [51].

As AI continues to expand in gastroenterology, particularly in treatment personalization and operational efficiency, ongoing rigorous research addressing these priorities will be crucial to unlocking AI’s full potential while safeguarding nursing values and patient care quality [14].

#### 4.6.2. Potential Future Applications Beyond the Included Evidence

None of the 11 included studies evaluated IBD flare prediction, nutrition risk scoring, symptom triage, wearable- or AI-integrated patient monitoring, sedation monitoring, instrument tracking, or adverse-event prediction in a gastroenterology nursing context. These applications are discussed only as plausible future directions suggested by the literature in adjacent specialties and adjacent AI-in-healthcare domains, and should not be read as findings of this scoping review. Prioritizing them for future nurse-led research and evaluation, alongside the evidence gaps identified in Section 3.5, would help close the gap between technically feasible AI applications and evidence of benefit to gastroenterology nursing practice.

### 4.7. Limitations of This Review

This review has several limitations. The search was restricted to English-language publications indexed in four databases, which may have excluded the relevant grey literature, non-English studies, or work indexed elsewhere. Synthesis was narrative rather than meta-analytic, reflecting the heterogeneity of included study designs, and no formal critical appraisal of study quality was reported (see Section 2.4). The evidence base itself is small (11 studies), geographically concentrated, and dominated by early-stage or single-center work, limiting the generalizability of the findings. Screening and data extraction were performed independently and in duplicate by two reviewers, with disagreements resolved through discussion. An additional, more fundamental limitation is that several included studies did not directly evaluate nursing practice or nurse-led AI implementation; in these cases, nursing relevance was inferred by the review team rather than measured by the original study authors (Table 3). This limitation bears directly on the strength of the review’s central claims and is arguably more consequential than the absence of formal critical appraisal.

## 5. Conclusions

The central finding of this review is that direct, nursing-specific evidence on AI in gastroenterology remains limited: most identified studies report on AI applications developed and evaluated primarily from a technical or physician perspective, with nursing relevance often indirect or inferred rather than directly measured. AI demonstrates considerable potential to support and enhance multiple facets of gastroenterology nursing practice, from diagnostics and patient monitoring to workflow optimization and personalized care. However, to realize these benefits fully, greater nurse involvement in AI development and evaluation is crucial. This involvement, coupled with rigorous real-world implementation research and ethical oversight, will ensure AI’s safe, equitable, and effective integration into clinical workflows.

A focused review of the intersection between AI, nursing, and gastroenterology is justified given the current scarcity of literature explicitly addressing this nexus, amid the growing importance of AI in transforming specialized nursing roles. Gastroenterology nursing is on the cusp of rapid innovation, driven by advances in AI technologies, digital communication tools, and increasing clinical specialization. Early evidence highlights improvements in care quality, efficiency, and patient satisfaction; nonetheless, ongoing research, comprehensive evaluation, and structured professional development remain essential to harness these opportunities responsibly.

This scoping review has synthesized the current literature exploring diverse AI and AI-adjacent digital technologies applied within gastroenterology nursing. Consistent with the central finding above, the evidence base remains small, geographically concentrated, and too rarely nurse-led to support firm conclusions about impact on nursing practice; further nurse-centred evaluation and implementation research—encompassing both quantitative and qualitative nursing data—is needed before the extent of AI’s contribution to gastroenterology nursing can be established. Given the overlap between nursing and other health disciplines, future reviews could expand scope to include broader health data systems, fostering interdisciplinary insights.

Overall, AI stands poised to transform gastroenterology nursing by advancing diagnostics, individualized patient monitoring, and care personalization. Yet, achieving successful adoption requires robust evidence backing, ethical governance, and comprehensive nurse education. Incorporating technological innovations, strategically prioritizing those that demonstrably improve clinical outcomes and nursing practice, is imperative. In this evolving landscape, nurses must commit to lifelong learning and adaptability to new technologies, ensuring competent and safe performance within an increasingly complex healthcare environment. Crucially, when evaluating new AI tools, a discerning approach is necessary to distinguish between simple automation and true clinical intelligence that meaningfully enhances patient care.

## Figures and Tables

**Table 2:** List of Authors per Year per Country.

Author	Year	Country
Wang, Y. et al. [38]	2022	Taiwan
Gu, F. et al. [39]	2024	China
Fu, Z. et al. [40]	2024	China
Zhao, Y. et al. [17]	2023	China
Garcia, P. et al. [36]	2024	United States
Wu, L. et al. [41]	2023	China
Marras, C. et al. [33]	2024	Spain
Manzari, Z.S. et al. [34]	2024	Iran
Garg, S.K. et al. [42]	2023	United States
Vanalli, M. et al. [37]	2023	Italy
Zhai, Y. et al. [43]	2024	China

**Table 3:** Eligibility-Criteria Check and Nursing-Involvement Classification per Included Study.

Author, Year	AI Criterion Met?	Gastroenterology Criterion Met?	Nursing Criterion	Nursing Involvement Level	Classification Notes
Wang, Y.; et al., 2022 [38]	Yes (CNN/deep learning)	Yes (colonoscopy)	Indirect	Level 4	Nursing role not directly explored or integrated into the study design.
Gu, F.; et al., 2024 [39]	Yes (ML ensemble: decision trees, SVM, XGBoost, BPN)	Yes (colonoscopy prep)	Indirect	Level 4	Nursing involvement described by the review team as “plausible”; not explicitly reported by the study.
Fu, Z.; et al., 2024 [40]	Yes (LLM + RAG)	Yes (digestive endoscopy nursing)	Direct	Level 2	Nurses are end-users and provided direct feedback on the tool.
Zhao, Y.; et al., 2023 [17]	Yes (NLP-based chatbot)	Yes	Direct	Level 2	Nurses use the chatbot directly to support patient communication and education.
Garcia, P.; et al., 2024 [36]	Yes (generative AI/LLM)	Yes (162 primary care and gastroenterology clinicians participated—corrected; see Table 1)	Direct	Level 2	Clinic nurses were among the direct users of the AI drafting tool, alongside physicians and advanced practice clinicians, within a gastroenterology-and-hepatology-inclusive care team; outcome data were not broken out by clinician type.
Wu, L.; et al., 2023 [41]	Yes (hybrid: rule-based/regex modules plus a deep-learning information-extraction model and NLP—corrected; see Table 1)	Yes (colorectal polypectomy follow-up)	Direct	Level 2	AI criterion met: the system combines rule-based logic with a deep-learning text-to-structure information-extraction (UIE) model and NLP. The trial directly measured a nursing-workload outcome: nurse time spent manually notifying patients fell from 2.86 h (105 patients) to 0 h with system assistance.
Marras, C.; et al., 2024 [33]	No (digital simulation trainer; no ML/AI component)	Partial (biosimilar-switching training, not GI-disease-specific)	Direct	Level 1	Nurses are primary users and participated in validation, but the tool itself is not AI.
Manzari, Z.S.; et al., 2024 [34]	No (study explicitly reports no advanced AI)	Yes (IBD patient education)	Direct	Level 1	Nurses are the educators/interventionists; AI criterion not met.
Garg, S.K.; et al., 2023 [42]	Partial (rule-based automated CDS in EHR)	Yes (colonoscopy sedation triage)	Direct	Level 2	Nursing staff directly operationalize the tool for sedation triage.
Vanalli, M.; et al., 2023 [37]	Yes (NLP + string-similarity mapping)	Partial (mixed medicine/neurology/gastroenterology units)	Direct	Level 1	Nursing documentation is the direct subject of the study.
Zhai, Y.; et al., 2024 [43]	Yes (ML: random forest, SVM, XGBoost)	Yes (cirrhosis)	Indirect	Level 3	Nursing relevance described as supporting risk stratification and care planning, not directly evaluated.

Nursing Involvement Level: Level 1, nurses as primary users of the AI (or AI-adjacent) tool; Level 2, nurses as intervention providers or direct end-users; Level 3, nurses indirectly affected by the application; Level 4, nursing implications inferred by the review team but not empirically evaluated by the primary study. Classification applied by the review team based on the descriptions in Table 1.

## Data Availability

Data sharing is not applicable to this article, as no new data were created or analyzed in this study. All data supporting this review are available in the cited published studies and in the Supplementary Materials (Files S1–S3).

## Supplementary Materials

The following supporting information can be downloaded at https://www.mdpi.com/article/10.3390/nursrep16100357/s1, File S1: PRISMA-ScR checklist; File S2: PubMed E-utilities reproducibility re-run (14 August 2026)—full query string and retrieved record list; File S3: Complete per-database search strategies.

## Public Involvement Statement

No public involvement in any aspect of this research.

## Guidelines and Standards Statement

This scoping review was drafted in accordance with the PRISMA-ScR (Preferred Reporting Items for Systematic Reviews and Meta-Analyses extension for Scoping Reviews) [35] for scoping review research; the completed checklist is provided as Supplementary File S1.
