# Supplementary material for: Artificial Intelligence and AI-Adjacent Digital Technologies in Nursing Practice Within Gastroenterology: A Scoping Review of Current Applications, Challenges, and Future Directions
Source: Nurs Rep. 2026 Oct 10;16(10):357. doi: 10.3390/nursrep16100357 (PMC13708654; doi:10.3390/nursrep16100357)
Supplement: Supplementary file 1 [file nursrep-16-00357-s001.zip › nursrep-4561182-supplementary-file S3.pdf]

Supplementary File S3: Complete per-database search strategies

This document details the exact search strings, controlled vocabularies, and parameters used to query the literature for this scoping review, ensuring complete reproducibility.

1. Search Parameters & Limits

- **Date Range:** January 1, 2000, to August 14, 2026.
- **Language Limit:** English language only.
- **MEDLINE Platform:** MEDLINE records were queried directly through the **PubMed-hosted platform** (Public E-utilities interface), meaning a separate Ovid-MEDLINE search was not required as the databases are cross-indexed on this platform.
- **Boolean & Truncation:** Standard Boolean operators (`AND`, `OR`) were used to combine concept blocks. The asterisk (`*`) was used for right-truncation to capture word variants (e.g., `nurse*` for nurse, nurses, nursing).

2. Per-Database Search Strings

The search concepts were broken down into three main blocks: Artificial Intelligence, Nursing, and Gastroenterology.

2.1 PubMed (including MEDLINE)

Search String:

```
("Artificial Intelligence"[MeSH Terms] OR "artificial intelligence"[Title/Abstract] OR "machine learning"[Title/Abstract] OR "deep learning"[Title/Abstract] OR "natural language processing"[Title/Abstract] OR "large language model*"[Title/Abstract]) AND ("Nursing"[MeSH Terms] OR "nursing"[Title/Abstract] OR "nurse*"[Title/Abstract] OR "nursing practice"[Title/Abstract]) AND ("Gastroenterology"[MeSH Terms] OR "gastroenterology"[Title/Abstract] OR "gastrointestinal"[Title/Abstract] OR "endoscopy"[Title/Abstract] OR "colonoscopy"[Title/Abstract])
```

Filters Applied: English, from 2000 - 2026.

2.2 Scopus

Search String:

```
TITLE-ABS-KEY ( ( "artificial intelligence" OR "machine learning" OR "deep learning" OR "natural language processing" OR "large language model*" ) AND ( nursing OR nurse* ) AND ( gastroenterology OR gastrointestinal OR endoscopy OR colonoscopy ) )
```

Filters Applied: PUBYEAR > 1999 AND (LIMIT-TO (LANGUAGE , "English")).

2.3 Web of Science (Core Collection)

Search String:

```
TS=( ( "artificial intelligence" OR "machine learning" OR "deep learning" OR "natural language processing" OR "large language model*" ) AND ( nursing OR nurse* ) AND ( gastroenterology OR gastrointestinal OR endoscopy OR colonoscopy ) )
```

Filters Applied: PY=(2000-2026) AND LA=(English).
