# Supplementary material for: Artificial Intelligence and AI-Adjacent Digital Technologies in Nursing Practice Within Gastroenterology: A Scoping Review of Current Applications, Challenges, and Future Directions
Source: Nurs Rep. 2026 Oct 10;16(10):357. doi: 10.3390/nursrep16100357 (PMC13708654; doi:10.3390/nursrep16100357)
Supplement: Supplementary file 1 [file nursrep-16-00357-s001.zip › nursrep-4561182-Supplementary_File_S1_PRISMA-ScR_Checklist.pdf]

## Supplementary File S1. PRISMA-ScR Checklist

*Artificial Intelligence and AI-Adjacent Digital Technologies in Nursing Practice Within Gastroenterology: A Scoping Review of Current Applications, Challenges, and Future Directions*

Completed checklist of the Preferred Reporting Items for Systematic Reviews and Meta-Analyses extension for Scoping Reviews (PRISMA-ScR) [Tricco et al., 2018; Ref. 35 of the main text]. The "Reported in" column gives the section, table, or figure of the main manuscript where each item is addressed. Checklist item wording is abridged from the original.

| Section | Item | PRISMA-ScR checklist item | Reported in |
|---|---|---|---|
| **TITLE** | | | |
| Title | 1 | Identify the report as a scoping review. | Title |
| **ABSTRACT** | | | |
| Structured summary | 2 | Provide a structured summary including, as applicable, background, objectives, eligibility criteria, sources of evidence, charting methods, results, and conclusions that relate to the review questions and objectives. | Abstract |
| **INTRODUCTION** | | | |
| Rationale | 3 | Describe the rationale for the review in the context of what is already known, and explain why the review questions/objectives lend themselves to a scoping review approach. | Section 1; Sections 1.1 and 1.3 |
| Objectives | 4 | Provide an explicit statement of the questions and objectives being addressed, with reference to their key elements (e.g., population or participants, concepts, and context). | Section 1.4; Section 2.1 |
| **METHODS** | | | |
| Protocol and registration | 5 | Indicate whether a review protocol exists, where it can be accessed, and, if available, registration information. | Section 2.4 (no protocol was registered in advance) |
| Eligibility criteria | 6 | Specify characteristics of the sources of evidence used as eligibility criteria (e.g., years considered, language, publication status), and provide a rationale. | Section 2.2 |
| Information sources | 7 | Describe all information sources in the search (e.g., databases with dates of coverage) and the date the most recent search was executed. | Section 2; Section 2.3; Supplementary File S3 |
| Search | 8 | Present the full electronic search strategy for at least one database, including any limits used, such that it could be repeated. | Section 2.3; Supplementary File S3 |
| Selection of sources of evidence | 9 | State the process for selecting sources of evidence (i.e., screening and eligibility) included in the review. | Section 2.2 |
| Data charting process | 10 | Describe the methods of charting data from the included sources of evidence, whether data charting was done independently or in duplicate, and any processes for obtaining and confirming data. | Sections 2.2 and 2.3; Section 4.7 |
| Data items | 11 | List and define all variables for which data were sought and any assumptions and simplifications made. | Section 2.3 |

| Section | Item | PRISMA-ScR checklist item | Reported in |
|---|---|---|---|
| Critical appraisal of individual sources of evidence | 12 | If done, provide a rationale for conducting a critical appraisal of included sources of evidence; describe the methods used and how this information was used in any data synthesis. | Section 2.4 (not performed; optional for scoping reviews) |
| Synthesis of results | 13 | Describe the methods of handling and summarizing the data that were charted. | Section 2; Section 2.3 |
| **RESULTS** | | | |
| Selection of sources of evidence | 14 | Give numbers of sources of evidence screened, assessed for eligibility, and included in the review, with reasons for exclusions at each stage, ideally using a flow diagram. | Section 3.1; Figure 5 |
| Characteristics of sources of evidence | 15 | For each source of evidence, present characteristics for which data were charted and provide the citations. | Section 3.2; Tables 1 and 2 |
| Critical appraisal within sources of evidence | 16 | If done, present data on critical appraisal of included sources of evidence. | Not applicable (Section 2.4); study-level limitations reported in Table 1 |
| Results of individual sources of evidence | 17 | For each included source of evidence, present the relevant data that were charted that relate to the review questions and objectives. | Tables 1 and 3 |
| Synthesis of results | 18 | Summarize and/or present the charting results as they relate to the review questions and objectives. | Sections 3.3–3.5 |
| **DISCUSSION** | | | |
| Summary of evidence | 19 | Summarize the main results (including an overview of concepts, themes, and types of evidence available), link to the review questions and objectives, and consider the relevance to key groups. | Sections 4.1–4.6 |
| Limitations | 20 | Discuss the limitations of the scoping review process. | Section 4.7 |
| Conclusions | 21 | Provide a general interpretation of the results with respect to the review questions and objectives, as well as potential implications and/or next steps. | Section 5 |
| **FUNDING** | | | |
| Funding | 22 | Describe sources of funding for the included sources of evidence, as well as sources of funding for the scoping review. Describe the role of the funders of the scoping review. | Funding statement (back matter); funding of included sources was not charted |

Reference: Tricco, A.C.; Lillie, E.; Zarin, W.; O'Brien, K.K.; Colquhoun, H.; Levac, D.; Moher, D.; Peters, M.D.J.; Horsley, T.; Weeks, L.; et al. PRISMA Extension for Scoping Reviews (PRISMA-ScR): Checklist and Explanation. Ann. Intern. Med. 2018, 169, 467–473. https://doi.org/10.7326/M18-0850.
