# Supplementary material for: Artificial Intelligence and AI-Adjacent Digital Technologies in Nursing Practice Within Gastroenterology: A Scoping Review of Current Applications, Challenges, and Future Directions
Source: Nurs Rep. 2026 Oct 10;16(10):357. doi: 10.3390/nursrep16100357 (PMC13708654; doi:10.3390/nursrep16100357)
Supplement: Supplementary file 1 [file nursrep-16-00357-s001.zip › nursrep-4561182-Supplementary_File_S2_PubMed_Reproducibility_Check.pdf]

Supplementary File S2

PubMed E-utilities reproducibility re-run (14 August 2026)—full query string and retrieved record list

Purpose

Section 3.1 and Figure 5 of the manuscript report the identification and screening figures from the review team's original search of PubMed, Medline, Web of Science, and Scopus. As an independent, dated check of the identification stage, this file documents a reproducible re-run of the search in the one source that does not require a subscription: PubMed, via its public E-utilities interface.

Search conducted

Database: PubMed (via NCBI E-utilities, esearch.fcgi / esummary.fcgi / efetch.fcgi).

Date run: 14 August 2026.

Query (constructed from the broad keyword strategy and filters described in Sections 2.2–2.3 — English language, published 2000 onward):

("artificial intelligence"[Title/Abstract] OR "artificial intelligence"[MeSH Terms]) AND (nursing[Title/Abstract] OR nurse[Title/Abstract] OR nurses[Title/Abstract] OR nursing[MeSH Terms]) AND (gastroenterology[Title/Abstract] OR gastrointestinal[Title/Abstract] OR endoscopy[Title/Abstract] OR colonoscopy[Title/Abstract] OR gastroenterology[MeSH Terms]) AND English[Language] AND ("2000"[Date - Publication] : "3000"[Date - Publication])

Result: 33 records (PMIDs listed in the table below). When re-checked on 8 October 2026, the same query restricted to records entered in PubMed up to 14 August 2026 (adding AND ("1900/01/01"[Date - Entrez] : "2026/08/14"[Date - Entrez])) returned the identical set of 33 PMIDs.

Cross-check against Table 1

The 33 retrieved records were compared by title and author against the 11 studies the manuscript reports as included (Table 1). Two direct matches were found: Garcia et al. (2024), "Artificial Intelligence-Generated Draft Replies to Patient Inbox Messages" (PMID 38506805), and Vanalli et al. (2023), "Natural language processing and String Metric-assisted Assessment of Semantic Heterogeneity..." (PMID 35403664) — both highlighted below. PubMed's record for Vanalli et al. lists the publisher DOI 10.7416/ai.2022.2517; because this DOI is not currently registered with Crossref, the reference list links to the PubMed record (PMID 35403664). The re-run also retrieved Lam et al. (2025) (PMID 40611396; Ref. 6 of the main text), which was assessed but not included, for the reasons given in Section 3.1.

The remaining 9 of the 11 included studies were not recovered by this single-database, keyword-only reproduction. This is expected and does not indicate an error in the original review: the original search used the database-specific strategies reported in Supplementary File S3 across all four databases (PubMed, Medline, Web of Science, and Scopus), whereas this check used a single keyword query in PubMed only. Scopus and Web of Science could not be queried for this check because they require institutional subscription access.

What this file does and does not establish

Does establish: that a plain, literal reading of the manuscript's own stated search strategy against a real, live, freely-accessible database returns a nonzero, checkable count, and that at least part of that count overlaps with the studies the authors report having included — a basic sanity check on the identification stage.

Does not establish: the authors' actual original per-database counts, duplicate counts, or title/abstract and full-text exclusion figures and reasons. Those require the original screening log and cannot be produced by an automated reproduction after the fact. They are reported, from the review team's screening records, in Section 3.1 and Figure 5 of the manuscript.

Retrieved records (PubMed, 14 August 2026)

#	PMID	Title	Year	Authors (first 3)
1	42546167	Artificial Intelligence-Automated Assessment of Colonoscopy Quality Metrics.	2026	Keswani RN, Kaklamanos E, Kristinsdottir K et al.
2	42528328	Development of a Machine Learning-Based Patient Classification System for a Gastroenterology Ward.	2026	Ren HF, Wei MF, Shen M et al.
3	42476531	Generating Patient Documents from Electronic Health Records Using Generative Artificial Intelligence: A Feasibility Study in a Japanese Cancer Center.	2026	Michihata N, Ishii H, Tsujimura H et al.
4	42314081	Development of a Knowledge Map for Managing Symptoms of Graft-Versus-Host Disease in Patients Undergoing Hematopoietic Stem Cell Transplantation.	2026	Shanshan L, Huili C, Mingxia C et al.
5	42032083	AI-assisted assessment of bowel preparation from patient-generated images for pre-procedure triage.	2026	Kim YC, Hwang H, Lee JB et al.
6	41919817	Precision Nursing: The Personalized Future of Care and Revolution in Clinical Practice.	2026	Lopes-Júnior LC
7	41647810	Asia-Pacific consensus on the use of artificial intelligence in colorectal cancer screening and surveillance.	2025	Koh FH, Li JW, Wong SH et al.
8	41604135	Two decades of artificial intelligence in colon cancer diagnosis and treatment: a bibliometric analysis of research trends (2003-2023).	2026	Wu CY, Zhong WJ, Wu SJ et al.
9	41587763	A Machine Learning Approach for Predicting Long-term Care Needs and Identifying Risk Factors Among Older Adults in Japan.	2025	Anezaki H, Hiroe M, Kawai M et al.
10	41450054	Predicting Postoperative Outcomes in Lower Gastrointestinal Surgery: A Machine Learning Approach Using Electronic Health Records.	2026	Chen LA, Wang SY, Lin SA
11	41199126	The application of machine learning in the evaluation of urinary tract infections incidence in patients in a Nursing and Treatment Facility.	2026	Grzegorzek U, Sobiak J, Jaworucka E et al.
12	41076017	The effect of autonomous mobile robots on improving endoscope reprocessing efficiency: a prospective comparative trial (with video).	2026	Shi Y, Bai J, Zhou M et al.
13	40973020	Gut Microbiota and Nutrition in Nursing Homes: Challenges and Translational Approaches for Healthy Aging.	2025	Jiménez-Arroyo C, Molinero N, Moreno-Arribas MV
14	40913795	The utility of artificial intelligence in gastrointestinal oncology: A systematic review of randomized controlled trials.	2025	Sorensen LE, Moore VA, Gregston JL et al.
15	40611396	A Model Predicting Artificial Intelligence Use by Gastroenterology Nurses in Clinical Practice: A Cross-Sectional Multicenter Survey. [Ref. 6; assessed, not included]	2025	Lam TYT, Hu Y, Yi Y et al.
16	40306978	British Society of Gastroenterology guidelines on colorectal surveillance in inflammatory bowel disease.	2025	East JE, Gordon M, Nigam GB et al.

#	PMID	Title	Year	Authors (first 3)
17	39910061	Eye-tracking dataset of endoscopist-AI teaming during colonoscopy: Retrospective and real-time acquisition.	2025	Zhu Y, Yang RJ, Fu PY et al.
18	39304088	Detection of Gastrointestinal Bleeding With Large Language Models to Aid Quality Improvement and Appropriate Reimbursement.	2025	Zheng NS, Keloth VK, You K et al.
19	39054913	Impact of User's Background Knowledge and Polyp Characteristics in Colonoscopy with Computer-Aided Detection.	2024	Lee J, Cho WS, Kim BS et al.
20	38561333	Information heterogeneity between progress notes by physicians and nurses for inpatients with digestive system diseases.	2024	Mashima Y, Tanigawa M, Yokoi H
21	38506805	**Artificial Intelligence-Generated Draft Replies to Patient Inbox Messages. [MATCHES Table 1]**	2024	Garcia P, Ma SP, Shah S et al.
22	41647953	Novel artificial intelligence-enabled deep learning system to enhance adenoma detection: a prospective randomized controlled study.	2023	Lachter J, Schlachter SC, Plowman RS et al.
23	36686571	Artificial intelligence empowers the second-observer strategy for colonoscopy: a randomized clinical trial.	2023	Wang P, Liu XG, Kang M et al.
24	35655733	Evaluation of Nursing Effects of Pelvic Floor Muscle Rehabilitation Exercise on Gastrointestinal Tract Rectal Cancer Patients Receiving Anus-preserving Operation by Intelligent Algorithm-based Magnetic Resonance Imaging.	2022	Zhang L, Wang F
25	35495895	Deep Learning-Based Ultrasound Combined with Gastroscope for the Diagnosis and Nursing of Upper Gastrointestinal Submucous Lesions.	2022	Xia L, Sun S, Dai W
26	35403664	**Natural language processing and String Metric-assisted Assessment of Semantic Heterogeneity method for capturing and standardizing unstructured nursing activities in a hospital setting: a retrospective study. [MATCHES Table 1]**	2023	Vanalli M, Cesare M, Cocchieri A et al.
27	35084542	Explainable artificial intelligence (XAI): closing the gap between image analysis and navigation in complex invasive diagnostic procedures.	2022	O'Sullivan S, Janssen M, Holzinger A et al.
28	35070023	When should we perform colonoscopy to increase the adenoma detection rate?	2021	Kim SH, Kim JH
29	34637147	Characterizing shared and distinct symptom clusters in common chronic conditions through natural language processing of nursing notes.	2021	Koleck TA, Topaz M, Tatonetti NP et al.
30	32998213	Frontiers of Robotic Gastroscopy: A Comprehensive Review of Robotic Gastroscopes and Technologies.	2020	Marlicz W, Ren X, Robertson A et al.
31	32990974	Robot-Assisted Total Pancreatectomy With Autologous Islet Cell Transplantation: Perioperative Nursing Considerations.	2020	Williams GM
32	31794063	Fifty years of digestive endoscopy: Successes, setbacks, solutions and the future.	2020	Axon ATR
33	30836800	Addressing priority challenges in the detection and assessment of colorectal polyps from capsule endoscopy and colonoscopy in colorectal cancer screening using machine learning.	2019	Blanes-Vidal V, Baatrup G, Nadimi ES
